# Supplementary material for: Plasmodium falciparum parasite prevalence in East Africa: Updating data for malaria stratification
Source: PLOS Glob Public Health. 2021 Dec 7;1(12):e0000014. doi: 10.1371/journal.pgph.0000014 (PMC7612417; doi:10.1371/journal.pgph.0000014)
Supplement: S1 Text — (DOCX) [file pgph.0000014.s001.docx]

**Supplementary Information 1: Data Acknowledgements**

Many institutions and individuals have generously provided data to be able to undertake the sub-regional malaria risk mapping and these are listed here.

***Research institutes and national malaria control programs who have provided archive assistance, access to national household survey data not in the public domain, data extraction and geo-coding***

The Kenyan Medical Research Institute (KEMRI) collaborative partnerships with the Wellcome Trust/University of Oxford, US Centers for Disease Control, Nagasaki University, Case-Western University and Walter Reed (Kenya), Eastern and Southern Africa Centre of International Parasite Control, KEMRI (Kenya), International Centre of Insect Physiology & Ecology (ICIPE) (Kenya), Kenya National Bureau of Statistics (KNBS); University of California San Francisco/Makerere University/Uganda Malaria Surveillance Project (UMSP) (Uganda), Infectious Diseases Research Centre (Uganda), Child Health & Development Centre, Makerere University (Uganda), Uganda Bureau of Statistics (UBOS); Ifakara Health Institute's collaborative partnerships with the Swiss Tropical Institute, London School of Hygiene and Tropical Medicine and US Centers for Disease Control (Tanzania), Dar es Salaam Urban Malaria Control Project (Tanzania), National Medical Research Institute (NMRI) (Tanzania), Tanzanian National Bureau of Statistics (TNBS) (Tanzania)

***Individuals who have provided finer temporal and spatial data from published works or have undertaken unpublished surveys and have generously provided community and school-level data.***

**Kenya:** Dorcas Akach, David Amadi, Joseph Anyona, Charles Arnold, Harrysone Atieli, Philip Bejon, Simon Brooker, Jon Cox, Meghna Desai, Mark Divall, Carol Gitonga, Katherine Halliday, Mary Hamel, Laura Hammitt, Jeremy Herren, Tobias Homan, Susan Imbahale, Rachel Jenkins, Elizabeth Juma, Jimmy Kahara, Alice Kamau, Simon Kariuki, David Kyalo, Stella Kepha, Christine Kerubo, Charles King, Rebecca Kiptui, Astrid Knoblauch, Feiko ter Kuile, Kayla Laserson, Tjalling Leenstra, Eugiena Lo, Brett Lowe, Hortance Manda, Damaris Matoke, Charles Mbogo, Sam Mbulaiteye, Margaret McKinnon, Noboru Minakawa, Richard Mukabana, Clifford Mutero, Charles Mwandawiro, Joseph Mwangangi, Lydia Mwangi, Tabitha Mwangi, Miriam Mwjame, Peter Ng’anga, Sammy Njenga, Patricia Njuguna, Pamela Nkatha, Abdisalan Mohamed Noor, Oscar Nyangari, Clair Null, George Nyangweso, Christopher Nyundo, Christopher Odero, Edna Ogada, Bernards Ogutu, Wendy Prudholme O’Meara, Collins Omondi Okoyo, Raymond Omollo, Collince J. Omondi, Monica Omondi, Daniel Onguru, Charles Opondo, Edward Otieno, Maureen Otinga, Milka Owuor, Norbert Peshu, Aaron Samuels, Antony Scott, Elses Simiyu, Bob Snow, Christine Stewart, Jennifer Stevenson, Parminder Suchdev, Willem Takken, Noriko Tamari, Constance Tenge, Feiko Ter Kuile, Emily Teshome, Abdoulie Touray, Juliana Wambua, Vincent Were, Nelli Westercamp, Guiyun Yan, Rahma Udu Yusu, Guofa Zhou

**Tanzania**: Evan Bloch, Frank Chacky, Prosper Chaki, Mercy Chiduo, Stephano Cosmas, Mark Divall, Lwidiko Edward, Saul Epimark, Filbert Francis, Joanna Gallay, Blaise Genton, Deus Ishengoma, Abdallah Kajuna, Esther Kawira, Rashid Khatib, Rose Kibe, Winifrida Kidima, Judy Kirama, Gerry Killeen, Pili Kimanga, Christopher Kisiringyo, William Kisinza, Immo Kleinshmidt, Astrid Knoblauch, Randall Kramer, Tom Lietman, Rose Lusinde, John Lusingu, Pendael Machafuko, Slanslaus Mafung’a, Julie Makani, Celine Mandara, Renata Mandike, Alpahaxar Manjurano, Nestory Masalu, Severa Massawe, Leonard Mboera, Sam Mbulaiteye, Witness Mchwampaka, Fidelis Mgohamwende, Sigsbert Mkude, Yeromin Mlacha, Bruno Mmbando, Ally Mohammed, Fabrizio Molteni, Dominic Mosha, Frank Mosha, Jackline Mosha, Grace Moshi, Elizabeth Msaki, Amina Msengwa, Beatriz Munoz, Doreen Mutemi, Dismas Mwalimu, Felister Mwingira, Billy Ngasala, Baraka Nzobo, Fredros Okumo, Milka Owuor, Natasha Protopopoff, Mark Rowland, Susan Rumisha, Bob Snow, Glades Stanley, Deborah Sumari, Sheila West, Jessica Lin

**Uganda**: Yap Boum, Teun Bousema, Ross Boyce, Clare Chandler, Jessica Cohen, Martin Donnelly, Grant Dorsey, Chris Drakeley, Thomas Egwang, Alison Elliot, Samuel Gonahasa, Francesco Grandesso, Janet Hemingway, Narcis Kabatereine, Moses Kamya, Susan Kasula, Henry Katamba, Ruth Kigozi, Simon Peter Kigozi, Yasin Kisambira, Allan Kisoma, Moses Kizza, Mary Kyohere, Geoff Lavoy, Ismail Dragon Legason, Steve Lindsay, Joseph Lewnard, Swaib Lule, Godfrey Magumba, Catherine Maiteki-Sebuguzi, Sam Mbulaiteye, Sam Mbulaiteye, Levi Mugenyi, Ronald Mulebeke, Peter Mutungi, Marjorie Najjengo, Maggie Nampijja, Joaniter Nankabirwa, Florence Nankya, Arthur Mpimbaza, Benigna Namara, Florence Nankya, Alex Ndyabakira, Jaffer Okiring, Peter Okui, Peter Olupot-Olupot, Sally Opus, Sunil Parikh, Tonny Owalla, Caesar Oyet, Carla Proietti, Andrea Rehman, Richard Sanya, Indrani Saran, Julius Sejjemba, Bob Snow, James Ssekitoleeko, Michelle Roh, Ronald Ssenyonga, Sarah Staedke, Claire Standley, Russell Stothard, Ambrose Talisuna, James Tibenderana, Emily Webb, Adoke Yeka
