# Supplementary material for: Plasmodium falciparum parasite prevalence in East Africa: Updating data for malaria stratification
Source: PLOS Glob Public Health. 2021 Dec 7;1(12):e0000014. doi: 10.1371/journal.pgph.0000014 (PMC7612417; doi:10.1371/journal.pgph.0000014)
Supplement: S2 Text — (DOCX) [file pgph.0000014.s002.docx]

**Supplementary Information 2: Covariate selection**

**Covariates**

A parsimonious set of covariates known to influence malaria transmission were assembled based on previous reviews [1-3], including:

*Precipitation*: The rate of annual precipitation data from 2000-2019 was obtained from the Climate Hazards Group InfraRed Precipitation with Stations (CHIRPS Version 2.0) (<https://data.chc.ucsb.edu/products/CHIRPS-2.0/>). CHIRPS-2.0 is archived at 0.05^o^ x 0.05^o^ spatial resolution produced after combining satellite imagery and in-situ station data to create gridded precipitation time series for trend analysis and seasonal drought monitoring [4].

*Enhanced Vegetation Index (EVI)*: Vegetation indices are used for global monitoring of vegetation conditions. EVI is a measure of photosynthetic activity ranging from 0 (no vegetation) to 1 (complete vegetation) was derived from Moderate-resolution Imaging Spectroradiometer (MODIS) sensor imagery (<http://modis.gsfc.nasa.gov/data/>) and summarized to produce a synoptic annual product (2000-2019).

*Urbanisation*: Nighttime lights were used to show the level of urbanicity/rurality and a proxy for socio-economic status [5]. Overall, malaria infection is usually lower in urban compared to rural areas of Africa because of reduced malaria vector density and biting rate [Hay et al., 2005]. Previous research has associated nighttime lights with population and human activity [Amaral et al., 2005]. Here, the nighttime lights were derived from DMSP-OLS (2000-2013) and Visible Infrared Imaging Radiometer Suite (VIIRS) (from 2013 - 2020) onboard the Suomi National Polar Partnership (NPP) satellite launched in 2011 with a spatial resolution of approximately 1 km at nadir. The data contain the mean of visible band digital number values of cloud-free light detections (<https://www.nasa.gov/nex/data>).

*Aridity Index (AI):* The AI expresses the degree of dryness of the climate at a given location. It derived as a generalized function of the ratio of annual precipitation to annual potential evapotranspiration (EP) [6, 7]. Soil moisture content, coupled with the soiling rate of drying reduces the availability of sites suitable for oviposition and reduces the survival of vectors at all stages of their development [8]. Here, the AI was obtained from (<https://cgiarcsi.community/data/global-aridity-and-pet-database/>) at approximately 1 km spatial resolution. The index was calculated using input data from WorldClim Global Climate data (<https://worldclim.org/>). The WorldClim data consist of a global geodatabase (with a spatial resolution of approximately 1km at the equator) of monthly mean data (1950-2000) for precipitation, mean, minimum and maximum temperature. AI = Mean annual precipitation / Mean Annual Potential Evapo-Transpiration

*Temperature Suitability Index (TSI)*: TSI was produced in 2011 as a measure representing the optimal *P. falciparum* sporozoite development at 1 × 1 km spatial resolution [9]. The TSI model uses a biological framework based on the survival of vectors and the fluctuating monthly ambient temperature effects on the duration of sporogony that must be completed within the lifetime of a single generation of Anophelines. The TSI is therefore a long-term monthly temperature time series and represented on a scale of increasing transmission suitability, from 0 (unsuitable) to 1 (most suitable).


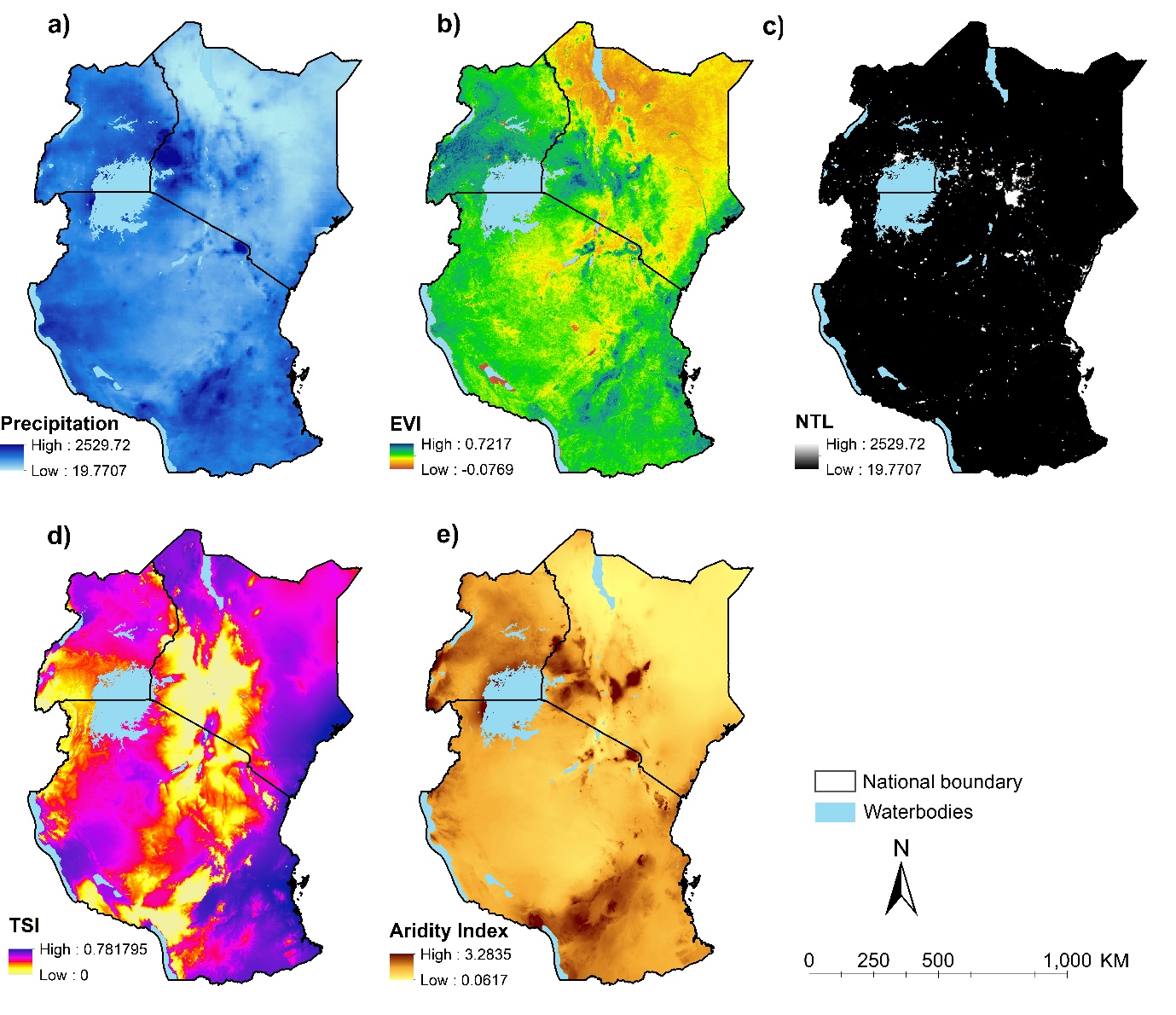


**Figure SI 2.1:** Maps of covariates at 1 × 1 Km spatial resolution showing: a) Annual mean precipitation; b) Annual mean enhanced vegetation index (EVI); c) Night-time lights (NTL); d) temperature suitability index; e) Aridity Index.

**Covariate selection**

A statistical procedure was used to select a minimum set of space-time covariates matching case data. This was necessary for avoiding statistical over-fitting [Babyak, 2004]. A subset of data where the sample size was ≥20 (*n*=7877) was selected from the assembled survey data and used for covariate testing. For each of the selected cluster, the extraction of covariates was carried out in ArcGIS 10.5 Spatial Analyst extension. 5 km buffers were generated at each cluster location and covariate means summarized for each location using focal statistics [10]. Covariates were standardized (scaled) using the observed mean and standard deviation.

Covariate selection was implemented in the *bestglm* package in *R* using the leap algorithm. Cross-validation (CV) approach was used where data was split into subsets and the best subset list selected based on a ten-fold cross-validation method. The parsimonious model corresponded to the model with the best CV score. Table SI 2.1 shows the statistical coefficient from covariate testing that yielded: precipitation, night-time light (NTL) and temperature suitability index (TSI) as the predictors. Figure SI 2.2 shows the decay in CV error based on input subset models.

**Table SI 2.1**: The results of the total-set analysis by country showing the regression coefficients and the P-values of the best-fit covariates.

|  | Coefficient | Std. Error | P value |
| --- | --- | --- | --- |
| Precipitation | 8.376 | 0.352 | <0.001 |
| TSI | 7.243 | 0.285 | <0.001 |
| NTL | -5.194 | 0.362 | <0.001 |


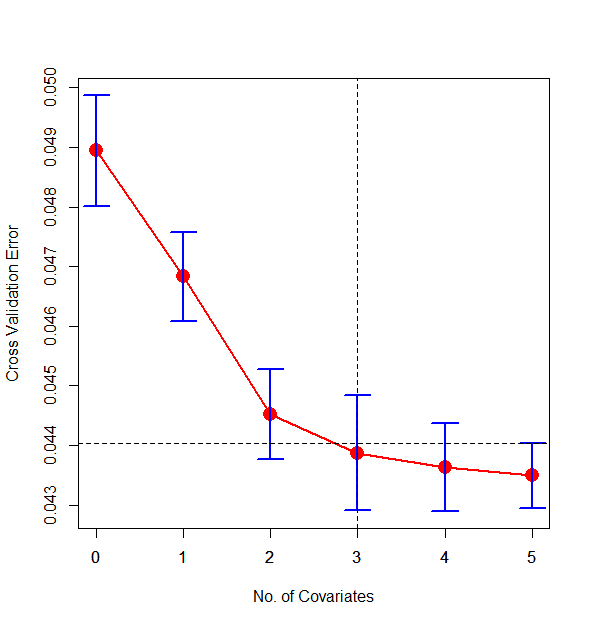


**Figure SI 2.2:** Model selection with 10-fold cross-validation and 1-sd rule with estimated cross-validation error in red across the number of covariates

**References**

1. Noor AM, Kinyoki DK, Mundia CW, Kabaria CW, Mutua JW, Alegana VA, et al. The changing risk of *Plasmodium falciparum* malaria infection in Africa: 2000-2013;10: a spatial and temporal analysis of transmission intensity. The Lancet. 2014;383(9930):1739-47. doi: 10.1016/S0140-6736(13)62566-0.

2. Weiss DJ, Mappin B, Dalrymple U, Bhatt S, Cameron E, Hay SI, et al. Re-examining environmental correlates of Plasmodium falciparum malaria endemicity: a data-intensive variable selection approach. Malaria Journal. 2015;14(1):68. doi: 10.1186/s12936-015-0574-x.

3. Odhiambo JN, Kalinda C, Macharia PM, Snow RW, Sartorius B. Spatial and spatio-temporal methods for mapping malaria risk: a systematic review. BMJ Global Health. 2020;5(10):e002919. doi: 10.1136/bmjgh-2020-002919.

4. Funk C, Peterson P, Landsfeld M, Pedreros D, Verdin J, Shukla S, et al. The climate hazards infrared precipitation with stations—a new environmental record for monitoring extremes. Scientific Data. 2015;2(1):150066. doi: 10.1038/sdata.2015.66.

5. Zhao M, Zhou Y, Li X, Cao W, He C, Yu B, et al. Applications of Satellite Remote Sensing of Nighttime Light Observations: Advances, Challenges, and Perspectives. Remote Sensing. 2019;11(17):1971. PubMed PMID: doi:10.3390/rs11171971.

6. Kimura R, Moriyama M. Determination by MODIS satellite-based methods of recent global trends in land surface aridity and degradation. Journal of Agricultural Meteorology. 2019;75(3):153-9. doi: 10.2480/agrmet.D-19-00003.

7. Sahin S. An aridity index defined by precipitation and specific humidity. Journal of Hydrology. 2012;444-445:199-208. doi: <https://doi.org/10.1016/j.jhydrol.2012.04.019>.

8. Shililu J, Ghebremeskel T, Seulu F, Mengistu S, Fekadu H, Zerom M, et al. Seasonal abundance, vector behavior, and malaria parasite transmission in Eritrea. J Am Mosq Control Assoc. 2004;20(2):155-64. Epub 2004/07/22. PubMed PMID: 15264625.

9. Gething PW, Van Boeckel TP, Smith DL, Guerra CA, Patil AP, Snow RW, et al. Modelling the global constraints of temperature on transmission of Plasmodium falciparum and P. vivax. Parasites & vectors. 2011;4(1):1-11.

10. Burgert CR. Spatial interpolation with Demographic and Health Survey data: Key considerations. Rockville, Maryland, USA: ICF International, 2014.
