# Supplementary material for: Plasmodium falciparum parasite prevalence in East Africa: Updating data for malaria stratification
Source: PLOS Glob Public Health. 2021 Dec 7;1(12):e0000014. doi: 10.1371/journal.pgph.0000014 (PMC7612417; doi:10.1371/journal.pgph.0000014)
Supplement: S3 Text — (DOCX) [file pgph.0000014.s003.docx]

**Supplementary Information 3****: Spatiotemporal modelling of malaria risk**

**Bayesian model specification**

A Bayesian hierarchical space-time model was implemented through a stochastic partial differential equations (SPDE) approach and using the Integrated Nested Laplace Approximations in R (R-INLA) [1, 2]. Bayesian inference was based on posterior distributions that combine data and appropriate prior knowledge (distributions) from model parameters via a likelihood function. The spatial effects introduced a measure of spatial autocorrelation in the model and thus, under Tobler’s first law of geography, clusters closer together in space would have *Pf*PR_2-10_ compared to those further apart in the spatial distance [3].

Let $s$ represent a set of cluster locations $n$ at which individuals tested for malaria. Then $y\left( s_{i},t \right)$ is the number of positive cases at time $t\left( t=1,....,T \right)$ with a Binomial likelihood and a linear predictor $\eta\left( s,t \right)$:

where $\eta\left( s,t \right)=\log it\left( P\left( s,t \right) \right)$ and the spatiotemporal process is defined as:

$\eta(s,t)=\beta_{0}+v(s,t)+e(s,t)$,

where $\beta_{0}$ is an intercept and for a generic location s, the process $v(s,t)$ is a mean-zero Spatio-temporal process (discrete yearly in the temporal domain) and $e(s,t)$ are i.i.d~$N\left( 0,\sigma_{e}^{2} \right)$. The error $e(s,t)$ was included as a residual adjustment to the spatio-temporal explanation. $v_{ij}(s,t)=Aw_{ij}(s,t)$, with $w_{ij}(s,t)\sim N(0,\Sigma)$ arising from a zero-mean Gaussian Matérn process with covariance $cov \left[ w(s,t),w(s',t') \right]\equiv C(s,s';t,t')$.

The spatial effect (spatial covariance) was modelled using the stochastic partial differential equation (SPDE) [4-6]. The spatio-temporal SPDE has the form:

$\left( k(s)^{2}-\Delta\right)^{\alpha/2}\left( \tau(s)x(s,t) \right)=w(s,t)$; $\alpha=\nu+d/2$;$k>0$,$v>0$

where $s$ denotes the spatial domain; $t$ the time domain; $\left( k(s)^{2}-\Delta\right)^{\alpha/2}$ is a differential operator; $k$ is the scaling parameter; $\Delta$ is the Laplacian; $\alpha$controls the smoothness of the realizations;$\tau$ controls the variance, $x(s,t)$ is the weight vector; and $w(s,t)$ is the Gaussian white noise. The precision matrix of the weight vector is generated as a joint spatial and temporal specification $Q= Q_{s}\bigotimes Q_{t}$ [7, 8] where $Q_{t}$ was based on auto-regressive parameter of first order AR(1).

The SPDE computation is achieved using a Gaussian Markov Random Function (GMRF) representation i.e. projecting the SPDE solution into local basis functions:

$$x(s,t)=\sum_{k=1}^{n} \phi_{k}(s,t)x_{k}$$

Where $x=(x_{1},\ldots\ldots,x_{n})∽N\left( \mu,Q^{-1} \right)$ is Markov for a neighbourhood structure $\left[ N_{i} i=1,\ldots,n \right]$ if $Q_{ij}=0$ when $j\neq N_{i}\cup_{i}$. This structure is realised by creating a Delaunay triangulation of the area of study. Each basis function is the Kronecker product $\phi_{k}(s,t)=\phi_{i}^{s}(s)\phi_{j}^{t}(t)$ in space and time. The advantage of using the GMRF representation stems from its Markovian properties that result in sparse precision matrices that are computationally efficient.

The SPDE use a Matérn covariance parametrised through a standard deviation $\sigma$, the spatial range parameter $\rho$ and a smoothing parameter.

$$C(r;\rho,\sigma)=\sigma^{2}\frac{2^{v-1}}{\Gamma(v)}\left( \sqrt{8\upsilon}\frac{r}{\rho} \right)^{v}k_{v}\left( \sqrt{8\upsilon}\frac{r}{\rho} \right)$$

where is the modified Bessel function of the second-order $\nu$ and is the marginal spatial variance. $\nu$ is the Matérn smoothness parameter linked to the spatial variance $\sigma^{2}$ through $\alpha=\nu+d/2$ where *d* is the spatial dimension. The more recent parameterisation of $\kappa=\sqrt{8\upsilon/\rho}$ and $\tau=\sigma\kappa^{\upsilon}\sqrt{\frac{\Gamma(v+d/2)\left( 4\pi\right)^{d/2}}{\Gamma(v)}}$ allow for construction is penalised complexity priors based on field range and standard deviation [9, 10]. Spatial specification similarly used PC prior approach with initial range parameter using a probability of 0.7 (i.e probability the spatial range was less than 50 km), and a standard deviation parameter of the spatial field not exceeding 1.

Let $\theta=\left\{ \beta,\sigma_{e}^{2},\kappa,\tau,\sigma_{w}^{2} \right\}$ represent a collection of unknown parameters (Figure SI 3.1). Then the joint posterior distribution is:

$$\theta\sim p\left( \theta\right)$$

$$x|\theta\sim N\left( 0,Q\left( \theta\right)^{-1} \right)$$

$$y_{i}|x,\theta\sim p\left( y_{i}|\eta_{i},\theta\right)$$

Where $y$ is the data, $\eta$ is the linear predictor, $x$ is the latent Gaussian field and $\theta$ the unknow hyper-parameters.

The Bayesian specification was completed by assigning zero-mean Gaussian prior distributions for the regression parameters such that$\beta_{j}\sim N(0,1/\tau_{\beta_{j}})$, the hyper-parameters for the temporal effect (year) as an autoregressive process of the first order $\xi_{ij}\sim N(0,1/\tau(1-\rho^{2}))$ and precision specified following penalised complexity (PC) prior [10].

**Figure SI 3.1:** Graphical representation of the mode showing parameter space of spatio-temporal regression.

**Constructing the mesh**

The objective of using SPDE approach is to find a GMRF that represent the Matérn field. Thus, finite element representation is used to define the Matérn field as a linear combination of basis functions defined on a triangulation of the domain. Figure SI 3.2 shows the mesh for East Africa countries. The guiding principles adopted for mesh was for inner triangles length to not exceeding model prior spatial range.


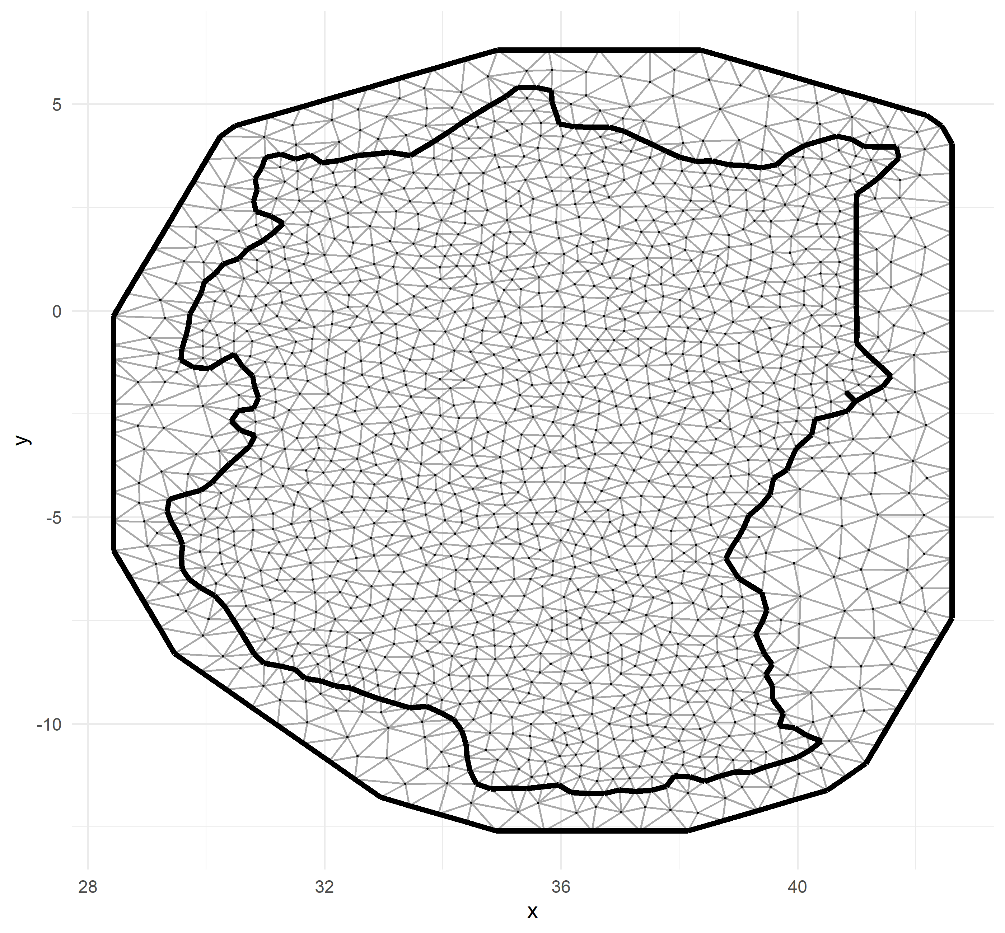


**Figure SI 3.2**: Constrained Refined Delaunay Triangulation for East Africa.

**Validation**

The *Pf*PR_2-10_ full space-time model validation statistics for the three countries (joint prediction validation statistics) for the model with and without covariates based on the actual observation and predictions to a 20% holdout data set.

MAE=mean absolute error; RMSE = the root mean square error; R= the linear correlation coefficient; MSE = the mean square error; and the DIC=Deviance Information Criterion. Note model with covariates used because of need for predictive modelling and explaining local variation of *Pf*PR_2-10_

| MAE | 0.02 |
| --- | --- |
| RMSE | 0.03 |
| R | 0.88 |
| MSE | 0.01 |
| DIC | 12911.82 |
| Marginal log-Likelihood | -10404.37 |

**References**

1. Rue H, Martino S, Chopin N. Approximate Bayesian inference for latent Gaussian models by using integrated nested Laplace approximations. Journal of the Royal Statistical Society: Series B (Statistical Methodology). 2009;71(2):319-92. doi: <https://doi.org/10.1111/j.1467-9868.2008.00700.x>.

2. Martins TG, Simpson D, Lindgren F, Rue H. Bayesian computing with INLA: New features. Computational Statistics & Data Analysis. 2013;67:68-83. doi: <https://doi.org/10.1016/j.csda.2013.04.014>.

3. Tobler W. Three presentations on geographical analysis and modeling: National Center for Geographic Information and Analysis. Santa Barbara, CA93106-4060: University of California, Santa Barbara, 1993.

4. Lindgren F, Rue H, Lindström J. An explicit link between Gaussian fields and Gaussian Markov random fields: the stochastic partial differential equation approach. Journal of the Royal Statistical Society: Series B (Statistical Methodology). 2011;73(4):423-98. doi: <https://doi.org/10.1111/j.1467-9868.2011.00777.x>.

5. Ingebrigtsen R, Lindgren F, Steinsland I. Spatial models with explanatory variables in the dependence structure. Spatial Statistics. 2014;8:20-38.

6. Lindgren F, Rue H. Bayesian Spatial and Spatio-temporal Modelling with R-INLA. Trondheim: Norwegian University of Science and Technology, Norway, 2013.

7. Simpson D, Lindgren F, Rue H. In order to make spatial statistics computationally feasible, we need to forget about the covariance function. Environmetrics. 2011;23(1):65-74. doi: 10.1002/env.1137.

8. Simpson D, Lindgren F, Rue H. Think continuous: Markovian Gaussian models in spatial statistics. Spatial Statistics. 2011;1(0):16-29.

9. Fuglstad G-A, Hem IG, Knight A, Rue H, Riebler A. Intuitive Joint Priors for Variance Parameters. Bayesian Anal. 2020;15(4):1109-37. doi: 10.1214/19-BA1185.

10. Fuglstad G-A, Simpson D, Lindgren F, Rue H. Constructing priors that penalize the complexity of gaussian random fields. J Am Stat Assoc. 2018:1-8. doi: 10.1080/01621459.2017.1415907.
