# Supplementary material for: Plasmodium falciparum parasite prevalence in East Africa: Updating data for malaria stratification
Source: PLOS Glob Public Health. 2021 Dec 7;1(12):e0000014. doi: 10.1371/journal.pgph.0000014 (PMC7612417; doi:10.1371/journal.pgph.0000014)
Supplement: S1 Table — (DOCX) [file pgph.0000014.s004.docx]

**Supporting Information 4:PA*Pf*PR_2-10_ Estimates**

| **ID** | **Country** |  | **Region** | **Admin 1** | **PAP*f*PR 2019** | **% of pop P*f*PR 90% NEP <1%** |
| --- | --- | --- | --- | --- | --- | --- |
| 1 | Kenya |  |  | Baringo | 0.92537 | 70.71 |
| 2 | Kenya |  |  | Bomet | 0.20809 | 94.74 |
| 3 | Kenya |  |  | Bungoma | 11.75704 | 7.77 |
| 4 | Kenya |  |  | Busia | 42.72191 | 0.00 |
| 5 | Kenya |  |  | Elgeyo-Marakwet | 0.62562 | 85.15 |
| 6 | Kenya |  |  | Embu | 0.08307 | 100.00 |
| 7 | Kenya |  |  | Garissa | 1.59915 | 79.26 |
| 8 | Kenya |  |  | Homa Bay | 6.28871 | 0.28 |
| 9 | Kenya |  |  | Isiolo | 0.33063 | 95.95 |
| 10 | Kenya |  |  | Kajiado | 0.25801 | 98.93 |
| 11 | Kenya |  |  | Kakamega | 21.58872 | 4.20 |
| 12 | Kenya |  |  | Kericho | 2.22634 | 39.53 |
| 13 | Kenya |  |  | Kiambu | 0.04320 | 100.00 |
| 14 | Kenya |  |  | Kilifi | 1.80424 | 28.12 |
| 15 | Kenya |  |  | Kirinyaga | 0.03912 | 100.00 |
| 16 | Kenya |  |  | Kisii | 2.28531 | 13.77 |
| 17 | Kenya |  |  | Kisumu | 21.85025 | 0.00 |
| 18 | Kenya |  |  | Kitui | 0.36887 | 99.36 |
| 19 | Kenya |  |  | Kwale | 6.05370 | 0.56 |
| 20 | Kenya |  |  | Laikipia | 0.10165 | 98.00 |
| 21 | Kenya |  |  | Lamu | 1.27578 | 67.42 |
| 22 | Kenya |  |  | Machakos | 0.07920 | 100.00 |
| 23 | Kenya |  |  | Makueni | 0.36392 | 97.15 |
| 24 | Kenya |  |  | Mandera | 0.73499 | 63.10 |
| 25 | Kenya |  |  | Marsabit | 0.68744 | 75.61 |
| 26 | Kenya |  |  | Meru | 0.15468 | 100.00 |
| 27 | Kenya |  |  | Migori | 16.84962 | 0.00 |
| 28 | Kenya |  |  | Mombasa | 8.19568 | 0.00 |
| 29 | Kenya |  |  | Murang'a | 0.03914 | 100.00 |
| 30 | Kenya |  |  | Nairobi | 0.10486 | 100.00 |
| 31 | Kenya |  |  | Nakuru | 0.08972 | 100.00 |
| 32 | Kenya |  |  | Nandi | 2.09913 | 38.86 |
| 33 | Kenya |  |  | Narok | 1.68334 | 74.98 |
| 34 | Kenya |  |  | Nyamira | 0.78743 | 70.66 |
| 35 | Kenya |  |  | Nyandarua | 0.00011 | 100.00 |
| 36 | Kenya |  |  | Nyeri | 0.01740 | 100.00 |
| 37 | Kenya |  |  | Samburu | 0.42570 | 96.06 |
| 38 | Kenya |  |  | Siaya | 45.80522 | 0.00 |
| 39 | Kenya |  |  | Taita Taveta | 0.41829 | 71.92 |
| 40 | Kenya |  |  | Tana River | 0.67814 | 47.02 |
| 41 | Kenya |  |  | Tharaka-Nithi | 0.16598 | 100.00 |
| 42 | Kenya |  |  | Trans Nzoia | 0.37748 | 83.72 |
| 43 | Kenya |  |  | Turkana | 4.81365 | 16.28 |
| 44 | Kenya |  |  | Uasin Gishu | 0.30840 | 93.65 |
| 45 | Kenya |  |  | Vihiga | 12.02692 | 0.00 |
| 46 | Kenya |  |  | Wajir | 0.63512 | 99.89 |
| 47 | Kenya |  |  | West Pokot | 1.98334 | 36.35 |
| 1 | Tanzania |  | Songwe | Songwe | 2.13989 | 2.07 |
| 2 | Tanzania |  | Manyara | Simanjiro | 0.20372 | 99.60 |
| 3 | Tanzania |  | Shinyanga | Shinyanga Rural | 9.30395 | 0.00 |
| 4 | Tanzania |  | Ruvuma | Namtumbo | 18.18890 | 0.02 |
| 5 | Tanzania |  | Kagera | Biharamulo | 33.29094 | 0.00 |
| 6 | Tanzania |  | Tabora | Sikonge | 5.57070 | 0.36 |
| 7 | Tanzania |  | Tanga | Tanga | 14.42537 | 0.00 |
| 8 | Tanzania |  | Manyara | Babati Urban | 0.02677 | 100.00 |
| 9 | Tanzania |  | Arusha | Arusha | 0.01064 | 100.00 |
| 10 | Tanzania |  | Kagera | Bukoba Rural | 17.25791 | 0.00 |
| 11 | Tanzania |  | Geita | Chato | 39.28106 | 0.00 |
| 12 | Tanzania |  | Mwanza | Ilemela | 5.78860 | 0.00 |
| 13 | Tanzania |  | Dar-es-salaam | Temeke | 4.65526 | 0.00 |
| 14 | Tanzania |  | Njombe | Makete | 0.02107 | 99.99 |
| 15 | Tanzania |  | Kilimanjaro | Same | 0.34653 | 99.98 |
| 16 | Tanzania |  | Arusha | Longido | 0.04141 | 100.00 |
| 17 | Tanzania |  | Kagera | Ngara | 22.96378 | 1.83 |
| 18 | Tanzania |  | Katavi | Mpimbwe | 6.42798 | 0.29 |
| 19 | Tanzania |  | Kilimanjaro | Siha | 0.00657 | 100.00 |
| 20 | Tanzania |  | Kagera | Muleba | 19.67374 | 0.58 |
| 21 | Tanzania |  | Morogoro | Ulanga | 21.43510 | 0.00 |
| 22 | Tanzania |  | Mbeya | Mbeya Rural | 1.93112 | 57.28 |
| 23 | Tanzania |  | Mbeya | Busokelo | 1.69675 | 52.43 |
| 24 | Tanzania |  | Iringa | Iringa Urban | 0.19657 | 100.00 |
| 25 | Tanzania |  | Shinyanga | Msalala | 28.14355 | 0.00 |
| 26 | Tanzania |  | Simiyu | Meatu | 0.67590 | 70.70 |
| 27 | Tanzania |  | Njombe | Njombe Urban | 0.04550 | 93.86 |
| 28 | Tanzania |  | Katavi | Mpanda Urban | 8.87872 | 0.00 |
| 29 | Tanzania |  | Tabora | Urambo | 28.58397 | 0.00 |
| 30 | Tanzania |  | Lindi | Liwale | 9.51052 | 1.43 |
| 31 | Tanzania |  | Morogoro | Malinyi | 16.77672 | 0.03 |
| 32 | Tanzania |  | Katavi | Mlele | 4.34314 | 10.19 |
| 33 | Tanzania |  | Shinyanga | Kishapu | 4.75057 | 17.27 |
| 34 | Tanzania |  | Njombe | Ludewa | 1.98188 | 64.25 |
| 35 | Tanzania |  | Pwani | Bagamoyo | 1.75945 | 61.96 |
| 36 | Tanzania |  | Pwani | Kisarawe | 13.52490 | 4.72 |
| 37 | Tanzania |  | Arusha | Meru | 0.01393 | 100.00 |
| 38 | Tanzania |  | Manyara | Mbulu | 0.02782 | 100.00 |
| 39 | Tanzania |  | Pwani | Mafia | 6.44364 | 0.00 |
| 40 | Tanzania |  | Mtwara | Newala | 23.48991 | 0.00 |
| 41 | Tanzania |  | Arusha | Ngorongoro | 0.05934 | 96.52 |
| 42 | Tanzania |  | Simiyu | Maswa | 7.52011 | 0.00 |
| 43 | Tanzania |  | Mtwara | Mtwara Urban | 20.72836 | 0.00 |
| 44 | Tanzania |  | Kigoma | Kigoma Rural | 17.63192 | 0.00 |
| 45 | Tanzania |  | Dodoma | Dodoma Urban | 0.13758 | 100.00 |
| 46 | Tanzania |  | Mbeya | Rungwe | 2.61342 | 59.06 |
| 47 | Tanzania |  | Tabora | Uyui | 22.01196 | 0.00 |
| 48 | Tanzania |  | Dodoma | Kongwa | 0.09496 | 99.96 |
| 49 | Tanzania |  | Mtwara | Nanyumbu | 19.66023 | 0.00 |
| 50 | Tanzania |  | Pwani | Chalinze | 9.38603 | 0.00 |
| 51 | Tanzania |  | Kigoma | Kakonko | 20.61778 | 0.00 |
| 52 | Tanzania |  | Tabora | Igunga | 7.12346 | 7.44 |
| 53 | Tanzania |  | Arusha | Karatu | 0.07330 | 100.00 |
| 54 | Tanzania |  | Arusha | Monduli | 0.01865 | 100.00 |
| 55 | Tanzania |  | Tanga | Muheza | 16.90301 | 3.17 |
| 56 | Tanzania |  | Mtwara | Newala TC | 13.02623 | 0.00 |
| 57 | Tanzania |  | Njombe | Njombe Rural | 0.81085 | 95.57 |
| 58 | Tanzania |  | Songwe | Ileje | 1.20986 | 27.22 |
| 59 | Tanzania |  | Dodoma | Bahi | 0.54999 | 95.41 |
| 60 | Tanzania |  | Mara | Bunda | 17.86412 | 0.00 |
| 61 | Tanzania |  | Katavi | Mpanda Rural | 26.05456 | 0.00 |
| 62 | Tanzania |  | Njombe | Makambako TC | 0.09231 | 100.00 |
| 63 | Tanzania |  | Kigoma | Uvinza | 23.36616 | 0.14 |
| 64 | Tanzania |  | Kilimanjaro | Hai | 0.02091 | 100.00 |
| 65 | Tanzania |  | Tanga | Pangani | 15.05469 | 0.00 |
| 66 | Tanzania |  | Mwanza | Sengerema | 26.49870 | 0.00 |
| 67 | Tanzania |  | Lindi | Mtama | 26.28345 | 0.00 |
| 68 | Tanzania |  | Shinyanga | Shinyanga Urban | 7.17374 | 0.00 |
| 69 | Tanzania |  | Mwanza | Misungwi | 19.85320 | 0.00 |
| 70 | Tanzania |  | Kigoma | Kibondo | 16.61800 | 0.04 |
| 71 | Tanzania |  | Iringa | Kilolo | 1.67320 | 84.42 |
| 72 | Tanzania |  | Singida | Iramba | 0.97027 | 78.53 |
| 73 | Tanzania |  | Lindi | Nachingwea | 11.02538 | 0.00 |
| 74 | Tanzania |  | Mara | Rorya | 22.29258 | 0.00 |
| 75 | Tanzania |  | Tabora | Nzega | 21.27267 | 0.00 |
| 76 | Tanzania |  | Singida | Ikungi | 1.33213 | 87.67 |
| 77 | Tanzania |  | Geita | Bukombe | 39.53985 | 0.00 |
| 78 | Tanzania |  | Morogoro | Mvomero | 6.44277 | 16.46 |
| 79 | Tanzania |  | Mtwara | Nanyamba | 39.77902 | 0.00 |
| 80 | Tanzania |  | Iringa | Mufindi | 0.24091 | 98.74 |
| 81 | Tanzania |  | Mwanza | Magu | 9.16880 | 0.07 |
| 82 | Tanzania |  | Morogoro | Kilombero | 14.24405 | 1.43 |
| 83 | Tanzania |  | Morogoro | Morogoro | 4.78596 | 10.60 |
| 84 | Tanzania |  | Mara | Tarime | 33.54296 | 0.00 |
| 85 | Tanzania |  | Mbeya | Chunya | 2.59989 | 7.48 |
| 86 | Tanzania |  | Mara | Musoma | 10.64020 | 0.00 |
| 87 | Tanzania |  | Kagera | Karagwe | 15.49892 | 6.56 |
| 88 | Tanzania |  | Simiyu | Bariadi | 6.91964 | 0.02 |
| 89 | Tanzania |  | Kigoma | Kasulu TC | 16.82914 | 0.00 |
| 90 | Tanzania |  | Tanga | Korogwe TC | 5.95196 | 0.00 |
| 91 | Tanzania |  | Lindi | Kilwa | 13.09342 | 1.31 |
| 92 | Tanzania |  | Tabora | Tabora MC | 16.07513 | 0.00 |
| 93 | Tanzania |  | Singida | Singida Urban | 0.04510 | 100.00 |
| 94 | Tanzania |  | Shinyanga | Kahama TC | 14.02789 | 0.00 |
| 95 | Tanzania |  | Shinyanga | Ushetu | 51.70469 | 0.00 |
| 96 | Tanzania |  | Mtwara | Masasi TC | 15.44084 | 0.00 |
| 97 | Tanzania |  | Singida | Singida | 0.02864 | 100.00 |
| 98 | Tanzania |  | Tanga | Handeni | 20.89316 | 0.01 |
| 99 | Tanzania |  | Singida | Mkalama | 0.13342 | 100.00 |
| 100 | Tanzania |  | Dar-es-salaam | Ilala | 2.90165 | 0.00 |
| 101 | Tanzania |  | Ruvuma | Nyasa | 6.99284 | 0.03 |
| 102 | Tanzania |  | Dar-es-salaam | Ubungo MC | 1.53132 | 4.86 |
| 103 | Tanzania |  | Kigoma | Kasulu | 31.28671 | 0.00 |
| 104 | Tanzania |  | Songwe | Mbozi | 0.49752 | 4.85 |
| 105 | Tanzania |  | Morogoro | Gairo | 0.84145 | 87.42 |
| 106 | Tanzania |  | Kilimanjaro | Moshi Municipal | 0.06326 | 100.00 |
| 107 | Tanzania |  | Simiyu | Bariadi TC | 5.10085 | 0.00 |
| 108 | Tanzania |  | Ruvuma | Mbinga | 3.68978 | 4.29 |
| 109 | Tanzania |  | Tabora | Kaliua | 28.00654 | 0.00 |
| 110 | Tanzania |  | Kigoma | Buhigwe | 15.52396 | 0.00 |
| 111 | Tanzania |  | Simiyu | Busega | 7.53797 | 0.00 |
| 112 | Tanzania |  | Ruvuma | Songea Urban | 2.04819 | 0.00 |
| 113 | Tanzania |  | Njombe | Wanging'ombe | 0.05593 | 100.00 |
| 114 | Tanzania |  | Songwe | Tunduma | 1.00069 | 0.00 |
| 115 | Tanzania |  | Arusha | Arusha Urban | 0.01222 | 100.00 |
| 116 | Tanzania |  | Tanga | Kilindi | 8.58630 | 6.04 |
| 117 | Tanzania |  | Mwanza | Buchosa | 36.35349 | 0.34 |
| 118 | Tanzania |  | Mwanza | Kwimba | 9.13833 | 0.00 |
| 119 | Tanzania |  | Morogoro | Morogoro Urban | 2.80015 | 1.11 |
| 120 | Tanzania |  | Iringa | Iringa Rural | 0.16725 | 99.96 |
| 121 | Tanzania |  | Mbeya | Mbarali | 0.48079 | 93.80 |
| 122 | Tanzania |  | Mbeya | Kyela | 7.17968 | 1.53 |
| 123 | Tanzania |  | Pwani | Kibaha | 6.06574 | 0.00 |
| 124 | Tanzania |  | Ruvuma | Tunduru | 27.23928 | 0.00 |
| 125 | Tanzania |  | Mara | Tarime TC | 33.42743 | 0.00 |
| 126 | Tanzania |  | Rukwa | Nkasi | 23.11522 | 0.00 |
| 127 | Tanzania |  | Ruvuma | Songea Rural | 9.41578 | 0.00 |
| 128 | Tanzania |  | Geita | Geita TC | 38.62357 | 0.00 |
| 129 | Tanzania |  | Dar-es-salaam | Kinondoni | 1.82380 | 2.60 |
| 130 | Tanzania |  | Kilimanjaro | Rombo | 0.04293 | 100.00 |
| 131 | Tanzania |  | Ruvuma | Mbinga TC | 1.12557 | 0.00 |
| 132 | Tanzania |  | Ruvuma | Madaba | 5.96652 | 23.55 |
| 133 | Tanzania |  | Manyara | Kiteto | 0.20183 | 93.77 |
| 134 | Tanzania |  | Dodoma | Chamwino | 0.22810 | 100.00 |
| 135 | Tanzania |  | Mara | Serengeti | 13.83812 | 0.34 |
| 136 | Tanzania |  | Mtwara | Masasi | 16.13832 | 0.00 |
| 137 | Tanzania |  | Rukwa | Sumbawanga MC | 0.94551 | 0.00 |
| 138 | Tanzania |  | Kagera | Missenyi | 14.48934 | 0.00 |
| 139 | Tanzania |  | Lindi | Ruangwa | 15.86393 | 0.00 |
| 140 | Tanzania |  | Kagera | Kyerwa | 9.34384 | 3.99 |
| 141 | Tanzania |  | Mbeya | Mbeya MC | 0.34383 | 97.84 |
| 142 | Tanzania |  | Tanga | Mkinga | 9.53057 | 0.00 |
| 143 | Tanzania |  | Kilimanjaro | Mwanga | 0.27161 | 100.00 |
| 144 | Tanzania |  | Mwanza | Ukerewe | 36.04025 | 0.04 |
| 145 | Tanzania |  | Tanga | Handeni TC | 17.20343 | 0.00 |
| 146 | Tanzania |  | Geita | Geita | 43.53180 | 0.00 |
| 147 | Tanzania |  | Mara | Musoma Municipal | 4.13982 | 0.22 |
| 148 | Tanzania |  | Mwanza | Nyamagana | 6.24045 | 0.00 |
| 149 | Tanzania |  | Pwani | Mkuranga | 23.06234 | 0.00 |
| 150 | Tanzania |  | Tanga | Korogwe | 2.80111 | 29.31 |
| 151 | Tanzania |  | Simiyu | Itilima | 3.28187 | 6.86 |
| 152 | Tanzania |  | Kagera | Bukoba Urban | 2.72911 | 0.00 |
| 153 | Tanzania |  | Kilimanjaro | Moshi | 0.06401 | 100.00 |
| 154 | Tanzania |  | Mara | Bunda TC | 12.43579 | 0.09 |
| 155 | Tanzania |  | Manyara | Hanang | 0.02226 | 100.00 |
| 156 | Tanzania |  | Iringa | Mafinga TC | 0.01504 | 100.00 |
| 157 | Tanzania |  | Morogoro | Kilosa | 5.75446 | 10.83 |
| 158 | Tanzania |  | Mara | Butiama | 17.59015 | 0.02 |
| 159 | Tanzania |  | Songwe | Momba | 6.81544 | 0.00 |
| 160 | Tanzania |  | Tanga | Lushoto | 1.02126 | 95.59 |
| 161 | Tanzania |  | Manyara | Mbulu TC | 0.00101 | 100.00 |
| 162 | Tanzania |  | Mtwara | Tandahimba | 30.88077 | 0.00 |
| 163 | Tanzania |  | Dodoma | Mpwapwa | 0.74481 | 79.43 |
| 164 | Tanzania |  | Kigoma | Kigoma MC-Ujiji | 21.45030 | 0.00 |
| 165 | Tanzania |  | Manyara | Babati | 0.04816 | 100.00 |
| 166 | Tanzania |  | Singida | Manyoni | 0.78941 | 80.82 |
| 167 | Tanzania |  | Tabora | Nzega TC | 6.83112 | 0.00 |
| 168 | Tanzania |  | Singida | Itigi | 0.92215 | 54.30 |
| 169 | Tanzania |  | Tanga | Bumbuli | 0.63572 | 84.84 |
| 170 | Tanzania |  | Pwani | Kibaha Urban | 1.71185 | 6.57 |
| 171 | Tanzania |  | Rukwa | Kalambo | 8.27741 | 0.00 |
| 172 | Tanzania |  | Dodoma | Chemba | 0.28148 | 96.03 |
| 173 | Tanzania |  | Geita | Mbogwe | 49.82921 | 0.00 |
| 174 | Tanzania |  | Geita | Nyang'wale | 41.21372 | 0.00 |
| 175 | Tanzania |  | Rukwa | Sumbawanga DC | 1.66871 | 0.00 |
| 176 | Tanzania |  | Katavi | Nsimbo | 14.63607 | 0.04 |
| 177 | Tanzania |  | Pwani | Rufiji | 5.18755 | 6.35 |
| 178 | Tanzania |  | Morogoro | Ifakara | 6.06443 | 1.82 |
| 179 | Tanzania |  | Lindi | Lindi Urban | 17.93563 | 0.00 |
| 180 | Tanzania |  | Mtwara | Mtwara Rural | 32.83072 | 0.00 |
| 181 | Tanzania |  | Dar-es-salaam | Kigamboni | 9.44178 | 0.00 |
| 182 | Tanzania |  | Pwani | Kibiti | 10.43247 | 4.39 |
| 183 | Tanzania |  | Dodoma | Kondoa Rural | 0.23526 | 100.00 |
| 184 | Tanzania |  | Dodoma | Kondoa Urban | 0.11405 | 100.00 |
| 1 | Uganda |  | Central | Masaka | 1.06126 | 71.69 |
| 2 | Uganda |  | Eastern | Butebo | 3.69320 | 0.00 |
| 3 | Uganda |  | Northern | Alebtong | 2.89501 | 0.00 |
| 4 | Uganda |  | Eastern | Bukedea | 8.89837 | 0.00 |
| 5 | Uganda |  | Eastern | Busia | 33.41206 | 0.00 |
| 6 | Uganda |  | Northern | Moroto | 9.49946 | 3.11 |
| 7 | Uganda |  | Western | Kabarole | 3.13270 | 3.77 |
| 8 | Uganda |  | Western | Masindi | 8.14867 | 0.15 |
| 9 | Uganda |  | Eastern | Kamuli | 24.72695 | 0.00 |
| 10 | Uganda |  | Eastern | Budaka | 1.74008 | 2.64 |
| 11 | Uganda |  | Northern | Maracha | 15.36044 | 0.00 |
| 12 | Uganda |  | Eastern | Tororo | 9.15029 | 0.00 |
| 13 | Uganda |  | Western | Kagadi | 9.84290 | 0.00 |
| 14 | Uganda |  | Central | Kalangala | 0.87815 | 78.86 |
| 15 | Uganda |  | Central | Buikwe | 13.08074 | 0.00 |
| 16 | Uganda |  | Central | Luwero | 3.81803 | 0.00 |
| 17 | Uganda |  | Northern | Kole | 23.64345 | 0.00 |
| 18 | Uganda |  | Northern | Amolatar | 8.03548 | 0.00 |
| 19 | Uganda |  | Northern | Lamwo | 12.17081 | 0.09 |
| 20 | Uganda |  | Western | Ibanda | 3.42652 | 16.01 |
| 21 | Uganda |  | Central | Wakiso | 1.90365 | 9.26 |
| 22 | Uganda |  | Northern | Kaabong | 21.90672 | 0.12 |
| 23 | Uganda |  | Western | Bunyangabu | 2.09926 | 13.78 |
| 24 | Uganda |  | Eastern | Iganga | 23.88628 | 0.00 |
| 25 | Uganda |  | Central | Nakasongola | 3.86355 | 0.00 |
| 26 | Uganda |  | Eastern | Kaberamaido | 1.52482 | 18.83 |
| 27 | Uganda |  | Eastern | Namayingo | 37.63727 | 0.00 |
| 28 | Uganda |  | Western | Rukungiri | 2.53180 | 37.13 |
| 29 | Uganda |  | Central | Mubende | 15.95371 | 0.00 |
| 30 | Uganda |  | Central | Buvuma | 7.70015 | 0.00 |
| 31 | Uganda |  | Northern | Pakwach | 16.79934 | 0.00 |
| 32 | Uganda |  | Western | Rubanda | 0.00095 | 99.89 |
| 33 | Uganda |  | Eastern | Luuka | 33.20982 | 0.00 |
| 34 | Uganda |  | Eastern | Soroti | 3.50572 | 0.00 |
| 35 | Uganda |  | Eastern | Kaliro | 21.16718 | 0.00 |
| 36 | Uganda |  | Northern | Omoro | 17.54187 | 0.00 |
| 37 | Uganda |  | Western | Rukiga | 0.03422 | 100.00 |
| 38 | Uganda |  | Eastern | Kween | 1.76500 | 42.50 |
| 39 | Uganda |  | Northern | Amuru | 16.88471 | 0.00 |
| 40 | Uganda |  | Northern | Kitgum | 9.61718 | 0.03 |
| 41 | Uganda |  | Western | Kyegegwa | 9.47780 | 0.00 |
| 42 | Uganda |  | Eastern | Serere | 6.73444 | 0.03 |
| 43 | Uganda |  | Western | Kanungu | 2.25560 | 16.87 |
| 44 | Uganda |  | Northern | Zombo | 13.11840 | 0.00 |
| 45 | Uganda |  | Eastern | Bukwo | 0.11453 | 53.28 |
| 46 | Uganda |  | Western | Kyenjojo | 5.74184 | 0.00 |
| 47 | Uganda |  | Eastern | Jinja | 35.68106 | 0.00 |
| 48 | Uganda |  | Eastern | Kapchorwa | 0.78339 | 73.05 |
| 49 | Uganda |  | Eastern | Butaleja | 2.57695 | 0.00 |
| 50 | Uganda |  | Eastern | Amuria | 6.54864 | 0.00 |
| 51 | Uganda |  | Western | Sheema | 0.40217 | 100.00 |
| 52 | Uganda |  | Northern | Yumbe | 14.13503 | 0.00 |
| 53 | Uganda |  | Western | Kasese | 3.00814 | 21.16 |
| 54 | Uganda |  | Western | Rubirizi | 3.92527 | 6.64 |
| 55 | Uganda |  | Northern | Adjumani | 12.86180 | 0.00 |
| 56 | Uganda |  | Eastern | Buyende | 21.32942 | 0.00 |
| 57 | Uganda |  | Western | Kisoro | 0.01457 | 98.43 |
| 58 | Uganda |  | Western | Ntungamo | 0.49219 | 83.71 |
| 59 | Uganda |  | Western | Mbarara | 0.35074 | 99.97 |
| 60 | Uganda |  | Northern | Nakapiripirit | 12.56588 | 6.76 |
| 61 | Uganda |  | Northern | Gulu | 17.57972 | 0.00 |
| 62 | Uganda |  | Central | Bukomansimbi | 1.14993 | 44.43 |
| 63 | Uganda |  | Western | Kibaale | 13.72896 | 0.00 |
| 64 | Uganda |  | Western | Kikuube | 7.72593 | 0.00 |
| 65 | Uganda |  | Central | Mpigi | 0.75450 | 57.71 |
| 66 | Uganda |  | Northern | Dokolo | 4.33693 | 5.40 |
| 67 | Uganda |  | Western | Mitooma | 1.19897 | 61.34 |
| 68 | Uganda |  | Central | Lwengo | 1.51198 | 9.19 |
| 69 | Uganda |  | Central | Kayunga | 18.16375 | 0.00 |
| 70 | Uganda |  | Central | Lyantonde | 0.79256 | 80.99 |
| 71 | Uganda |  | Central | Ssembabule | 1.40154 | 43.63 |
| 72 | Uganda |  | Eastern | Bududa | 0.92143 | 48.33 |
| 73 | Uganda |  | Northern | Agago | 4.56588 | 0.00 |
| 74 | Uganda |  | Northern | Apac | 38.52015 | 0.00 |
| 75 | Uganda |  | Eastern | Pallisa | 5.75495 | 0.00 |
| 76 | Uganda |  | Northern | Lira | 7.31524 | 0.00 |
| 77 | Uganda |  | Northern | Kotido | 29.26572 | 0.00 |
| 78 | Uganda |  | Central | Butambala | 0.89115 | 42.83 |
| 79 | Uganda |  | Central | Kalungu | 1.12458 | 53.09 |
| 80 | Uganda |  | Eastern | Katakwi | 9.94445 | 0.00 |
| 81 | Uganda |  | Western | Kiruhura | 0.67605 | 87.31 |
| 82 | Uganda |  | Western | Buliisa | 8.92129 | 0.00 |
| 83 | Uganda |  | Western | Kamwenge | 5.49742 | 0.00 |
| 84 | Uganda |  | Eastern | Manafwa | 8.68613 | 0.00 |
| 85 | Uganda |  | Central | Kyankwanzi | 4.25596 | 0.00 |
| 86 | Uganda |  | Northern | Napak | 18.37222 | 1.03 |
| 87 | Uganda |  | Western | Buhweju | 0.48084 | 93.90 |
| 88 | Uganda |  | Eastern | Namutumba | 9.78397 | 0.00 |
| 89 | Uganda |  | Central | Kiboga | 2.66723 | 1.93 |
| 90 | Uganda |  | Eastern | Bugiri | 14.33505 | 0.00 |
| 91 | Uganda |  | Northern | Otuke | 4.52418 | 0.00 |
| 92 | Uganda |  | Eastern | Kumi | 8.89073 | 0.00 |
| 93 | Uganda |  | Northern | Arua | 18.76029 | 0.00 |
| 94 | Uganda |  | Eastern | Sironko | 2.32753 | 26.87 |
| 95 | Uganda |  | Northern | Nebbi | 16.36778 | 0.00 |
| 96 | Uganda |  | Central | Kampala | 3.22598 | 0.00 |
| 97 | Uganda |  | Northern | Pader | 5.45480 | 0.00 |
| 98 | Uganda |  | Eastern | Mbale | 3.51065 | 6.73 |
| 99 | Uganda |  | Western | Kakumiro | 13.37202 | 0.00 |
| 100 | Uganda |  | Northern | Abim | 10.75540 | 0.00 |
| 101 | Uganda |  | Eastern | Ngora | 5.58208 | 0.00 |
| 102 | Uganda |  | Central | Mukono | 12.90807 | 0.00 |
| 103 | Uganda |  | Central | Kyotera | 4.76657 | 0.00 |
| 104 | Uganda |  | Western | Kabale | 0.00152 | 100.00 |
| 105 | Uganda |  | Northern | Amudat | 7.43337 | 0.00 |
| 106 | Uganda |  | Eastern | Mayuge | 29.65832 | 0.00 |
| 107 | Uganda |  | Western | Bushenyi | 1.16062 | 86.18 |
| 108 | Uganda |  | Central | Nakaseke | 1.78969 | 0.27 |
| 109 | Uganda |  | Central | Mityana | 1.70235 | 1.43 |
| 110 | Uganda |  | Western | Isingiro | 2.63145 | 25.07 |
| 111 | Uganda |  | Western | Ntoroko | 8.91644 | 2.68 |
| 112 | Uganda |  | Northern | Nwoya | 20.19569 | 0.00 |
| 113 | Uganda |  | Western | Bundibugyo | 13.69239 | 14.10 |
| 114 | Uganda |  | Western | Kiryandongo | 26.40578 | 0.00 |
| 115 | Uganda |  | Eastern | Namisindwa | 8.04452 | 4.98 |
| 116 | Uganda |  | Northern | Oyam | 31.35299 | 0.00 |
| 117 | Uganda |  | Northern | Koboko | 15.44022 | 0.00 |
| 118 | Uganda |  | Eastern | Bulambuli | 3.91204 | 43.85 |
| 119 | Uganda |  | Central | Gomba | 2.57166 | 0.00 |
| 120 | Uganda |  | Central | Rakai | 3.22312 | 0.04 |
| 121 | Uganda |  | Northern | Moyo | 8.46972 | 0.00 |
| 122 | Uganda |  | Eastern | Kibuku | 3.62854 | 0.00 |
| 123 | Uganda |  | Central | Kassanda | 4.27317 | 0.00 |
| 124 | Uganda |  | Western | Hoima | 3.18612 | 0.00 |
| 125 | Uganda |  | Northern | Kwania | 35.62371 | 0.00 |
| 126 | Uganda |  | Eastern | Kapelebyong | 5.99340 | 0.00 |
| 127 | Uganda |  | Northern | Nabilatuk | 24.14759 | 0.00 |
| 128 | Uganda |  | Eastern | Bugweri | 14.91968 | 0.00 |
| 129 | Uganda |  | Western | Rwampara | 0.35462 | 98.85 |
| 130 | Uganda |  | Western | Kitagwenda | 8.24599 | 3.28 |
| 131 | Uganda |  | Northern | Madi Okollo | 21.29142 | 0.00 |
| 132 | Uganda |  | Northern | Karenga | 17.71496 | 0.10 |
| 133 | Uganda |  | Northern | Obongi | 12.92728 | 0.00 |
| 134 | Uganda |  | Eastern | Kalaki | 1.53345 | 7.56 |
| 135 | Uganda |  | Western | Kazo | 2.64697 | 3.24 |
