# Supplementary material for: Plasmodium falciparum parasite prevalence in East Africa: Updating data for malaria stratification
Source: PLOS Glob Public Health. 2021 Dec 7;1(12):e0000014. doi: 10.1371/journal.pgph.0000014 (PMC7612417; doi:10.1371/journal.pgph.0000014)
Supplement: S1 Fig — (DOCX) [file pgph.0000014.s005.docx]

**Supplementary Information 5: Additional maps**


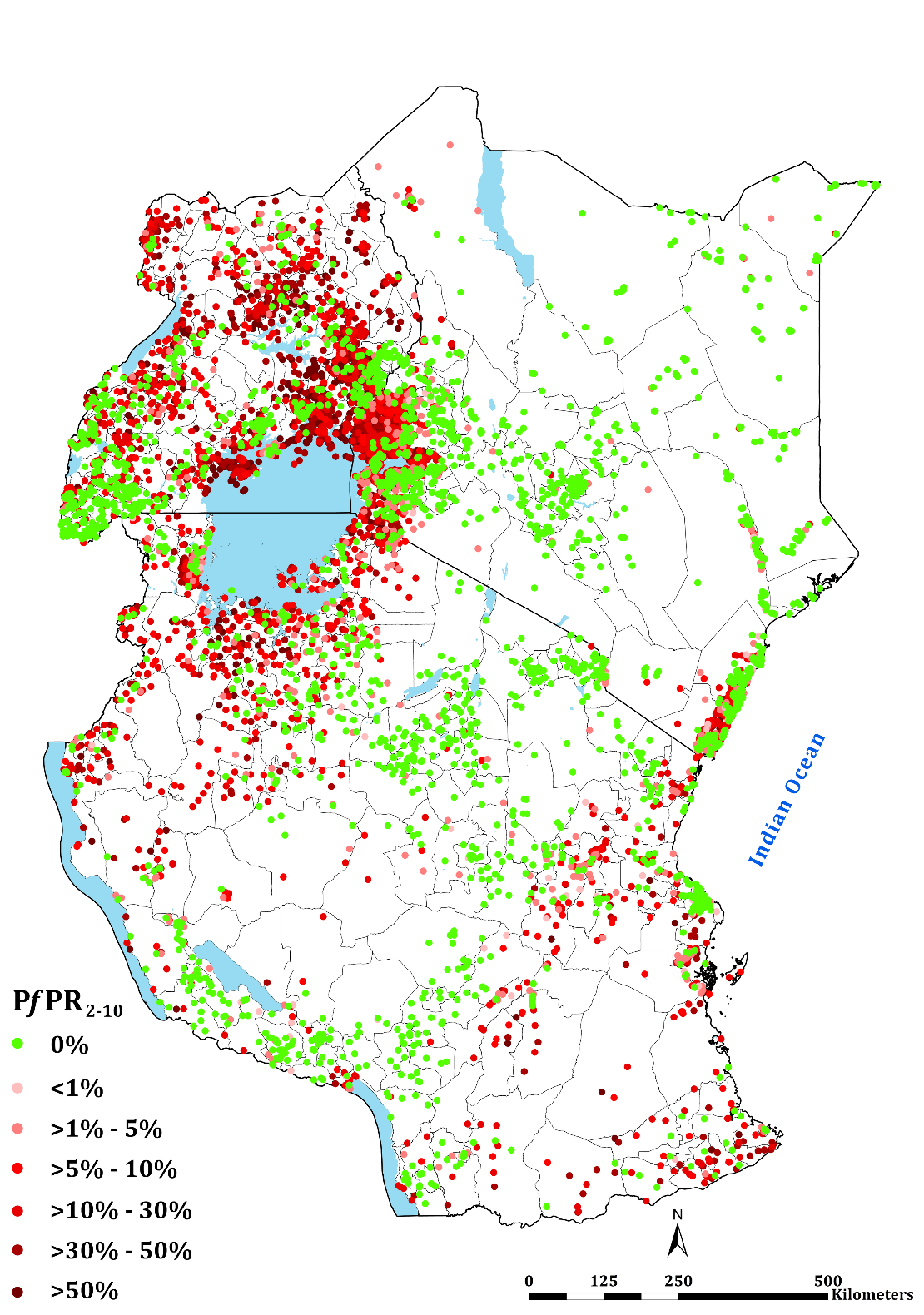


**Figure S5.1:**Distribution of all assembles survey data (n=18,941) between 2010-2020 of age-corrected and microscopy-standard parasite prevalence (PfPR_2-10_) estimates among samples ≥10 individuals with the lowest value shown on top.


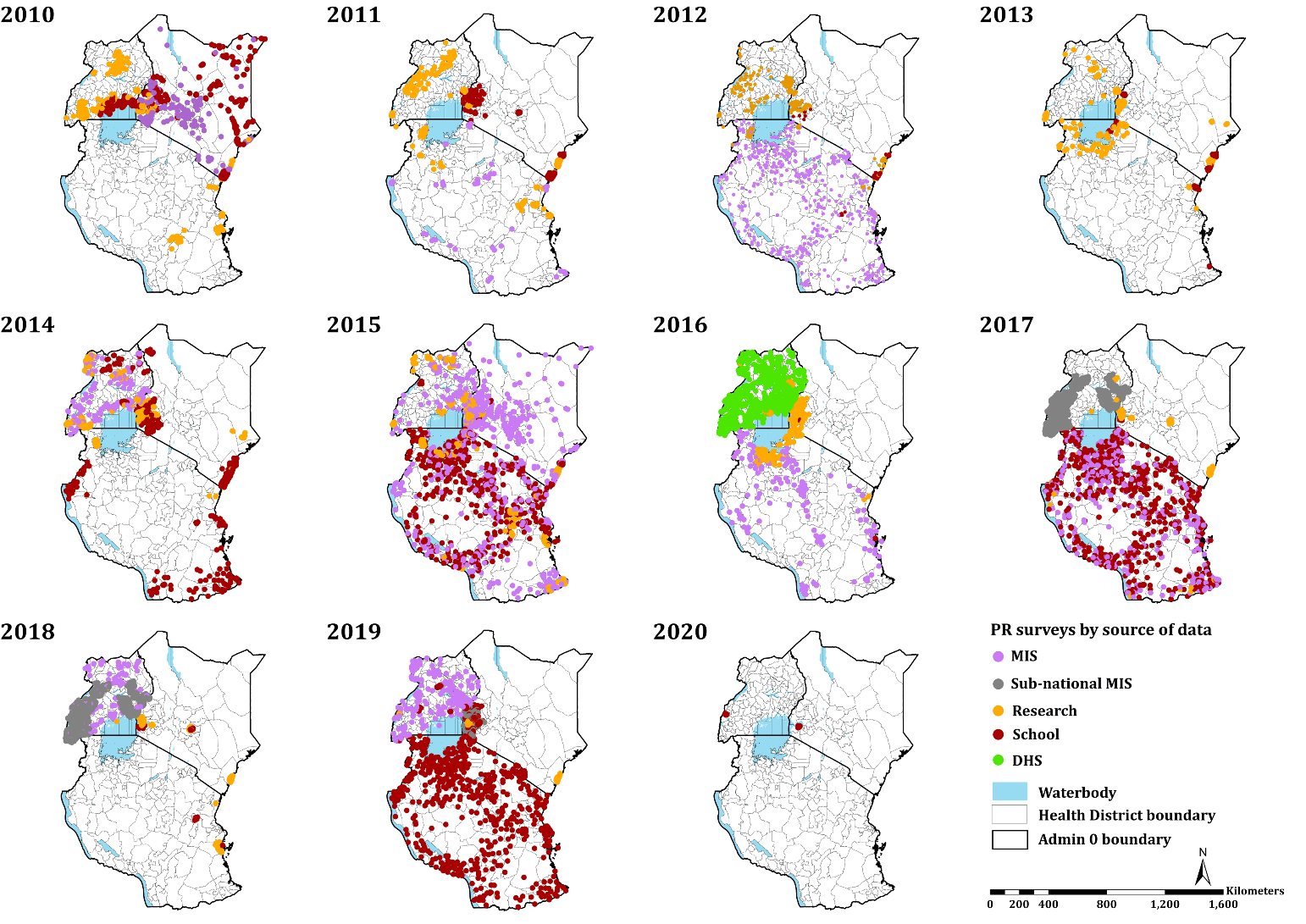


**Figure S5.2:** Distribution of surveys 2010-2020 according to the source of data.


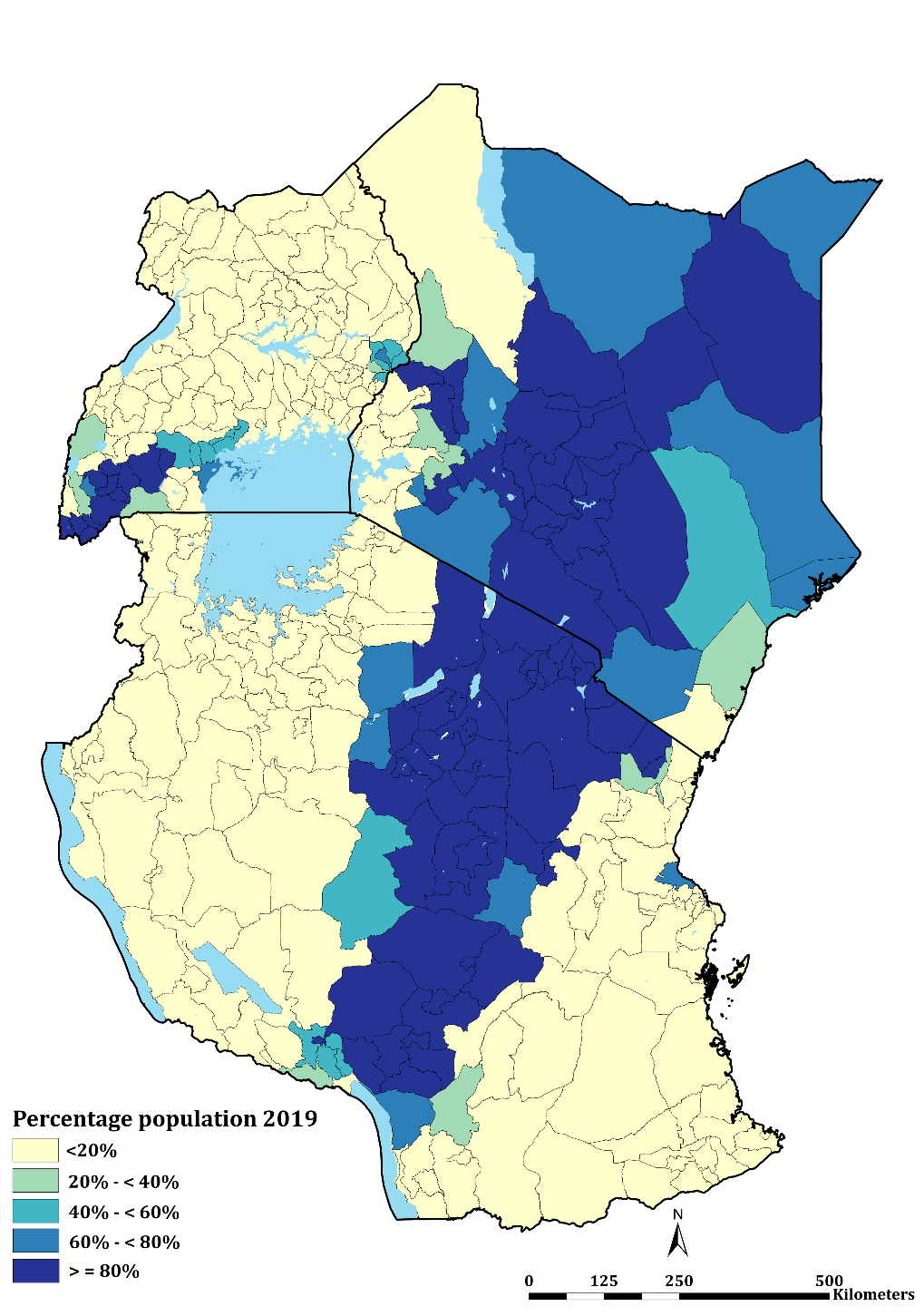


**Figure S5.3:** Estimated percentage population based on (PAPfPR_2-10_) 90% certain to be <1% (Non-exceedance probability (NEP)) by health district.
