# Supplementary material for: Plasmodium falciparum parasite prevalence in East Africa: Updating data for malaria stratification
Source: PLOS Glob Public Health. 2021 Dec 7;1(12):e0000014. doi: 10.1371/journal.pgph.0000014 (PMC7612417; doi:10.1371/journal.pgph.0000014)
Supplement: Attachment — Submitted filename: Response_to_reviewers_letter_VAA_010921.docx [file pgph.0000014.s006.docx]

Ref: PGPH-D-21-00103

Corresponding author

Alegana Victor

Email: [valegana@kemri-wellcome.org](mailto:valegana@kemri-wellcome.org)

August 30, 2020

*PLOS Global Public Health*

**Re: Plasmodium falciparum parasite prevalence in East Africa: updating data for malaria stratification**

Dear Editor,

We would like to thank you and the reviewers for the positive comments that overall, have improves the quality of the manuscript. We have made revisions as follows:

**Editorial comments**

**Regarding reviewer 2's comment on data availability, please confirm if the survey data since 2017 is expected to be included in your data repository, and the justification for release of this data being postponed until 2025. Note that the** [**PLOS Data policy**](https://journals.plos.org/climate/s/data-availability) **requires authors to make all data underlying the findings described in their manuscript fully available without restriction, with rare exception.**

*Response: We are deeply committed to Open Access data, releasing 115 years of malaria survey data from across Africa on Harvard Dataverse in 2017. We are equally committed to building national data ownership within African National Malaria Control Programmes (NMCPs). This was our priority under INFORM project, aimed at supporting NMCPs in the region.*

*There is an unfortunate tendency for national malaria data to be found only at overseas research institute sites. We are trying to change this paradigm by building NMCP capacities to curate, own and disseminate data from their own country/program. To this end, 3 months ago, we have shared the geo-coded data with each respective NMCP (Kenya, Uganda and Tanzania mainland). We have indicated how the general public might request these data from each NMCP and feel this is entirely legitimate and in line with PLOS policy. This data is available without restrictions and only need to be requested from NMCP along with other data hosted by MoH. We have added additional information in the data access section on how this data can be requested and who should be contacted among the respective NMCP co-authors.*

*Most reviewers have highlighted the growing importance of routine DHIS2 data for malaria stratification. We agree and address these issues below. However, with respect to data access, permission to use national DHIS2 must be granted by the relevant ministry of health. Our approach to the prevalence data is the same that data access to be channelled to respective ministry of health who are also co-authoring this work. In same analogy PLOS have published research using routine data hosted by various MoH or provided email address of individuals that can be contacted (e.g. (Plotkin et al., 2018, Ouma et al., 2021).*

*Finally, with respect to the updating of the Harvard DataVerse legacy database since 1900, we do plan to update this periodically. The first iteration was a result of 25 years of data searching, curation and geo-coding. Every 5-10 years we will repeat this exercise, but its relevance for the present paper and data access has always been superseded by our comments above and we have therefore deleted this section in the Data Access section which now reads*

“As part of a continued commitment by the KEMRI-Wellcome Trust’s INFORM programme (http://inform-malaria.org/) to support sub-regional National Malaria Control Programs, the final geo-coded prevalence databases have been provided to the respective Ministries of Health to be integrated into national malaria data repositories. Data sharing and access is the responsibility of national governments and data requests can be directed to NMCPs. Kenya ( EO – [eloyugi@gmail.com](mailto:eloyugi@gmail.com) & <http://www.nmcp.or.ke/index.php/contact-us>); Tanzania (FC – [chackyfa@gmail.com](mailto:chackyfa@gmail.com) & <https://www.moh.go.tz/en/contact-us> ); and Uganda (DR – [damianamanya@gmail.com](mailto:damianamanya@gmail.com) & <https://www.health.go.ug/contact-us/>).”

**Journal Requirements:
Please review your reference list to ensure that it is complete and correct. If you have cited papers that have been retracted, please include the rationale for doing so in the manuscript text, or remove these references and replace them with relevant current references. Any changes to the reference list should be mentioned in the rebuttal letter that accompanies your revised manuscript. If you need to cite a retracted article, indicate the article’s retracted status in the References list and also include a citation and full reference for the retraction notice.**

*Response: Only 4 references have been added (Reference 64, 65, 88 and 96)*

**Reviewer #1**

**Overall: This analysis involved a large data collection effort to produce an analysis of programmatic relevance. The outputs are clear and operationally relevant. Some methodological decisions could use some further justification and areas of the results and discussion could be expanded to complete the picture of how this analysis – and others like it to come – may be used by programs for strategy decisions.

Major comments:
1. Clarify the abstract**

*Response: We thank the reviewer for useful suggestions to improve the manuscript. We have now revised the entire manuscript and the abstract by improving sentence structure, logical flow and where possible followed suggestion from the first review of the article.*

**2. Methods – some additional justification for how the limited set of covariates were selected from the three references cited would be useful. There could be additional land cover, potential evaporation index, etc variables of interest. The covariates with the most positive predictive power were identified, but were methods explored to identify the dominant covariates in different operational units? That type of information would be useful for programs.**

*Response: A qualitative rather than quantitative methodology was used to select the most useful covariates. This was largely based on previous knowledge, experience and modelling we have conducted in Africa before and carefully considering best approaches while using covariates (for example in (Noor et al., 2014) we carefully selected covariates in each county and similar approaches in (Noor et al., 2009, Alegana et al., 2013, Alegana et al., 2016, Macharia et al., 2018, Giorgi et al., 2021).*

*More importantly, Weiss et al., 2015 reviewed a more extensive set of covariates quantitively for modelling PR that leveraged covariates and included interaction terms in sSA. We did not repeat this quantitative modelling but used the result of that study to qualitatively select covariates that would be predictive of parasite prevalence. In the study by Weiss et al., 2015, there were 20 covariate terms selected from an initial 923 untransformed covariates. Of these 15 variables characterized temperature, and 4 characterized vegetation index (EVI), land cover class, slope, and precipitation. These broad areas provided a guide in the selection of broad area of covariates for use in modelling parasite prevalence. However, we did not compute interactions of these covariate mix because we set out to conduct predictive modelling. It is not clear how increasing complexity e.g. by including interaction terms would improve model predictive performance without introducing (a) collinearity and (b) regression overfitting. For example, including both landcover and EVI introduces collinearity.*

*In our recent study (Giorgi et al., 2021) using Tanzania as a case study, we assembled and tested this broad set (Population density, night-time light, Precipitation, Temperature, Enhanced Vegetation Index) for predictive and explanatory modelling of parasite prevalence testing overfitting and collinearity. This study concluded that from this broad representation, some covariates could not improve both sensitivity and specificity of the model and a more parsimonious model was better identifiable. We used results from this study to conduct present covariate selection and we have cited this work in main paper.*

*As the purpose was to conduct predictive modelling, we zoned covariates at a cluster level rather than at operation administrative level. Comparing covariates at operational units would be useful if small area is of interest with aim of smoothing estimates across different districts see (Godwin and Wakefield, 2021).*

*Finally, the national malaria programs (NMPs) in these three countries do not yet have the capacity to conduct these complex modelling (last paragraph of the discussion).Information on covariates is not new to NMPs not just in East Africa but across sub-Saharan Africa. Indeed some countries still rely of climate-driven maps to characterise malaria risk (Alegana et al., 2020).*

*The assembly, selection and statistical analysis of parasite prevalence is not trivial and NMCPs do not have human resource nor computational tools to be able to conduct this at present. The ideal scenario is that NMCPs in future are able to do this or have dashboards that would facilitate such analysis.*

**More information on how time was used – time of survey and matching to the time of covariates would be useful – were monthly covariates and/or lagged variables used?**

*Response: We used long term covariates for prediction or representative period when no new covariate data is available e.g. 2011 TSI. We did not lag these covariates because that will be useful while characterising seasonality.*

*We recognise that using long-term mean covariates or static covariates is not representative of transmission season - inter and intra annual variation. We have now included this as a limitation in the discussion. Additionally, we clarify*

a) *We did not assess for seasonal variation. Seasonal mapping of parasite prevalence requires additional data sampling each month and matching such observation to monthly covariates. Additional sampling is depended on additional funding to NMCPs. Surveys are cross-sectional and conducted only for few months of the year (e.g. July in Kenya). Thereafter, experiments will need to be performed on model sensitivity and specificity with additional temporal data cube.*
b) *Seasonal mapping including lagging covariates is best conducted using routine health facility data (page 23 of the discussion). It will be appropriate to match routine monthly data from passive surveillance to monthly covariates to characterise risk by month. However, there are limitations for using health facility data as outlined on page 23 of the discussion. For example, hybrid approaches are required to pair community surveys (sampling in asymptomatic population) with health facilities data representing symptomatic population. Such approaches are currently under-developed (Page 23 of the discussion).*
c) *In the methods section, we state that we used long-term means of covariates that have been used in previous studies on mapping parasite prevalence (Snow et al., 2017, Macharia et al., 2018)*
d) *The input data were based on cross-sectional studies undertaken at different time of the year. Attempting to link single survey data to monthly covariates will increase model complexity by increasing unknowns where no data is available (see supplementary 5 additional maps). It is also not clear how new clusters will be generated at a national level in months or year where there is no data or where Admin 1 is missing data. Including covariates for period without data will increase model complexity (absence of data observations) and often contribute to regression overfitting. To avoid this overly complex model structure we resolved data to long-term mean over period targeted for prediction.*

**3.** **Results – it would be useful to have more presentation of the certainty of the predictions in the results section. Perhaps the non-exceedance map here and an exceedance map as well.**

*Response: The Exceedance map is opposite of map non-exceedance probability map presented in Figure 5. Indeed, areas where _PfPR2-10_ exceed 1% are shown in the map. We have therefore not added further maps.*

**4. Discussion – it would be interesting to have a bit more information about how these outputs could be used alongside the routine data that programs are familiar with and rely on. In spite of the biases of routine data – as you mention – there are many advantages to it like learning the seasonal signals of transmission.**

*Response: We agree there are many advantages of using routine data as highlighted on page 23.*

*The objective was not to show how this data layer presented in this manuscript could be used alongside other data (Routine data). Indeed, this layer should be used alongside other data layers (routine, vector, LLIN distributions, resistance etc)*

*As outline in the manuscript, we aimed to provide this important layer that can be used for stratification. Surprisingly, research on pairing data from asymptomatic population with symptomatic population presenting at health facility is underdeveloped. It would be inappropriate to speculated how that can be done without carefully assembled data in each population mix.*

**Minor comments:
1. Abstract – in the introduction, it would be good to introduce that you are providing a modeled output and not simply presenting the PR data available (as you do at the end of the introduction). With your modeled outputs there are stratification values beyond the sampled areas.
In the methods section, the wording of data being “undertaken” is a bit confusing. Perhaps say something like, “Malaria prevalence data from surveys undertaken between January 2010 and June 2020 were assembled from each of the three countries”.**

*Response: Revised*

**Do you adjust the data by the population, enviro, and eco covariates? Or did you simply use them as covariates in the modeling workflow?**

*Response: Population layer was used to estimate populations at risk. Environmental covariates were used for predictive modelling.*

**Conclusion – I am curious how some of the additional data types listed would help increase the use of prevalence data? PR data seem independent of some of these (for example, care seeking). Would stratification be more useful of these additional metrics were accounted for alongside PR data?**

*Response: Stratification should improve significantly if this other metrics are used alongside predicted parasite prevalence. For example, combining PfPR_2-10_ with malaria care-seeking behaviour could optimize deployment of LLINs routinely compared to assuming care seeking is uniform across different malaria endemicities. Additionally, combining PfPR_2-10_ with malaria vector distribution will inform some of vector control strategies e.g. Where IRS may be cost effective for indoor-biting vectors. These two approaches can then be combined to improve stratification shown in the manuscript. However, we did not assemble these additional data.*

**2. Introduction – in the abstract you mention the Global Technical Strategy and here HBHI. Is the use of multiple metrics for planning quoted in both strategies?**

*Response: Revised abstract to match the introduction.*

**What do you mean by vulnerability in the first paragraph? Like environmental receptivity? Or the impact of resource scarcity/vulnerability on different populations?**

*Response: We have deleted the word vulnerabilities to remove ambiguity without changing the meaning of sentence. We agree it could refer to both unavailability of resources as well as environmental receptivity.*

**Paragraph 2 – are surveys not powered to support sub-national stratification? Or is that they lack complete geographic coverage? If it’s both – then those would be two separate issues worth mentioning.**

*Response: Yes, the two sentence mentions this fundamental problem of surveys “*…these data have been collected from national household surveys undertaken every 3-5 years such as the Demographic and Health Surveys (DHS) or Malaria Indicator Surveys (MIS). However, because they lack adequate power for effective sub-national stratification on their own…”.

*We refer the readers to reference 9 in the manuscript and we have not added further text to the two sentences apart from pointing out the DHS and MIS.*

*To explain here, surveys are not powered on biomarkers i.e. to detect difference in parasite prevalence at cluster level. The precision required to achieve such an objective would require larger surveys that are more costly than the available funding for NMCPs in malaria endemic countries. The MIS is powered on malaria intervention indicators (Long Lasting Insecticide Treated nets) while DHS are powered on maternal health indicators for women of childbearing age – family planning – Reference MIS/DHS manuals but analysis in (Alegana et al., 2017).*

**Not sure ‘interpolation’ is the right to say what MBG was used for
Great to see the list of countries with country-specific prevalence maps. Does the first list use PR data?**

*Response: Interpolation is the correct technique for MBG methods. Basically, the model predictions are interpolation within the domain of the data frame i.e. there are not extrapolated although there are some boundary conditions that are technically dealt with during modelling (Banerjee et al., 2004).*

*Yes – the first list of countries uses PR data we have added the word “parasite prevalence to improve clarity “…*Modelled national parasite prevalence maps have been developed to inform sub-national approaches to control in Kenya*…”*

**3. Methods – the geographic scope and context section seems to belong in the introduction section.**

*Response: We disagree, this section describes the study area which should be in the methods sections. We have therefore not moved this section to introduction*

**Parasite data survey data assembly – it would be useful to have citations for what national PR survey sources are included in the existing data sources [ref 58].**

*Response: We have now edited this section to improve clarity on the listed data sets and their sources. This included data previously published in reference 58 and updated with new data since 2017. Since the publication of 2017 data, the notable improvements is geo-referencing of clusters based on EA sampling frames.*

*We have not described data since 1900 because: (a) we have not used that data in the present analysis and (b) these data were published in 2017 (Snow et al., 2017)*

**Is that what paragraph 3 of this section outlines? Or we these included in addition from what is in reference 58? The first sentence of the third paragraph in this section seems like a list – are you saying that these are surveys that were gathered?**

*Response: Correct, paragraph 3 is a complete description of data that has been used in present study. These include old and new surveys that were repeated post 2017 and independent surveys listed in this paragraph.*

**Great to list the updates and improvements made to the 2017 open access data. What resolution had the DHS and MIS data been at previously? Not the cluster level? The regional level?**

*Response: The DHS are standardized and representative of broad geographic regions. DHS are stratified at a national level by regions (e.g. Admin 1 in Kenya – as 8 Provinces) and by urban and rural (cluster classified as urban or rural). There is no further stratification. These means that the survey is representative of these broad regions e.g. national or regional mean can be estimated without loss of precision. However, estimate at the cluster level can be computed or interpolated. The cluster however comprises of only a small sample of household within the locality.*

**For the study location – lat/longs were given for point observations I assume, but how were areal units like EAs treated? Was the centroid used? Or the whole area?**

*Response: EA centroid was calculated. We have clarified in the manuscript*

**Were the survey data matched to covariates by month? Year? How were areal units matched?**

*Response: Firstly, areal data were not used. An EA centroid was established, and covariates extracted based on the Lat/Long.*

*Secondly, covariates were not matched to the month (see above explanation)*

**Prediction resolutions adjusted for population density – was zero risk given to pixels with 0 TSI and 0 population or either or?**

*Response: Pixels with zero population were assigned zero risk because it would be unreasonable to estimate parasite prevalence in areas uninhabited. This irrespective of temperature unsuitability (TSI=0). This was then followed by areas where temperature was unsuitable (TSI =0).*

**Was there any consideration for comparing the WorldPop data to the CIESEN population data (**[**https://data.humdata.org/dataset/highresolutionpopulationdensitymaps**](https://data.humdata.org/dataset/highresolutionpopulationdensitymaps)**)?**

*Response: We did not aim to compare different population density maps within this analysis. We simply selected and used the most popular population resource for disease mapping (Tatem et al., 2012). Worldpop is also adjusted to match the UN population totals at a national level. Therefore, the total population at a national level matches UN estimates.*

*We however acknowledge that different population maps could result in different population at risk as pointed out in “the effects of population choice on estimates of population at risk (Tatem et al., 2011)”*

**4. Results – an impressive amount of new data assembled since the 2017 publication! Great to see.**

**For Figure 1 – the reds in the colour scale used for B and C are hard to tell apart.**

*Response: We have changed Figure 1 to include only Figure 1A and Figure 1B. We have now included Figure 1C in the supplementary information 5 – additional Figures/maps*

**Figure 2 is great. It would be useful to see – maybe in an SI – how the colors shown in Fig 2B would map spatially.**

*Response: Done. We have created an additional supplementary information 5 (SI 5) for additional figures/maps.*

**Figure 3 – are the very low PR areas in W Tanzania parks/game reserves? Is that an artifact of the population surface?**

*Response: Yes, this is an artifact of population*

**Figure 4 and Table 1 are great for programmatic use. Would be great to see what you have in SI 4 as a map as well.**

*Response: Done, SI 5*

**5. Discussion – are the 18,000+ surveys assembled since 2010 for this region specifically?
What about limitations with the methods? Aside from the data only? And data limitations beyond the survey data? Is the TSI surface from 2011? Issues with the population data?**

*Response: We agree there were some limitations related to covariates such as static TSI (e.g. TSI was produced in 2011). We have added this limitation in the discussion on page 23.*

*We have also added the limitation of providing intra annual seasonal risk using cross-sectional parasite prevalence data.*

*The need for improved sampling in very low risk areas using school surveys is outlined on page 23, of the discussion.*

*Population maps are annually projected, and countries conduct a census every 10 years. This is unlikely to change by 2023 when mapping is likely to be repeated.*

**Reviewer #2**

**The paper is essentially an update to the previous malaria prevalence maps published by the same group across the East Africa region. The overall goal of this work is to feed into the malaria risk stratification planning that has been highlighted by the WHO as critical piece to help with targeting intervention mixes appropriately and monitor the progress of malaria burden. This paper highlights that prevalence estimates are simply one of the key surfaces in the grand scheme that is stratification, but not the final outcome, and should be used appropriately in conjunction with other burden metrics that the countries collect. Whilst the methodology nor data may not be ground-breaking it is certainly an important piece of work operationally. I don’t have major comments besides one:

1. Whilst I commend that the authors have shared these data back to their respective ministries of health and agree that work should be country led, it strikes me as odd to not update your own published data repository for up to four years – Why? This seems to not be in line with the authors key message of transparency and neither fits with the journal’s ethics of data sharing.**

*Response: We have responded comprehensively as part of editorial comment from the first submission.*

*We are committed to open data sharing. For this manuscript, we have outline how the general public can access data and provide email addresses of people that can be contacted from NMCPs in the data sharing section. We hope that this improves clarity for data access and is inline with the journal publication policy of ensuring access without any restriction.*

*As for Harvard Dataverse, we aim to update this periodically every 5-10 years. The first update occurred after 25 years of data assembly.*

**Minor
1. Introduction – authors suggest that there is lack of consistency in epidemiological stratification within the region, however, this paragraph is counterintuitive to their original premise of a ‘one-size-fits-all’ approach defined by the WHO will not be sufficient – consider revising this paragraph**

*Response: Revised by removing the word consensus from the sentence – i.e. The HBHI encourages countries to use multiple data and conduct risk stratification, tailoring interventions in various risk strata.*

**2. Limitation of including averaged environmental covariates for a long temporal band (2010 – 2020) is worth acknowledging in the discussion. Using an averaged of the environment is not representation of the biological system of transmission.**

*Response: Revised with this submission (page 23). Future mapping could improve resolving of covariate to year e.g. remodeling TSI annually or monthly with increased sampling of PR.*

**3. The data included here from DHS is GPS displaced in urban areas by 2km and in rural up to 5km which can mean that prediction outputs are mis-specified. Whilst there isn’t much you can do I think it should be acknowledged in the discussion section about the DHS.**

*Response: In methods section, page 8 we state: “*Geographic coordinates of the individual-level survey and national household survey data were revised using village coordinates from the national statistical agency geo-coded village or EA databases, updated school databases, population settlement shapefiles from the Ministries of Lands, Google Maps and research surveys geolocated using Global Positioning Systems (GPS).*”*

*Therefore, we did not use the displaced coordinates because we had access to survey sampling frames.*

**Figures
1. Figure 1 – figure 1A seems unnecessary and can be removed (though the legend can be consolidated with the PfPR legend).**

**Figure 1C also doesn’t provide the reader much information and can be moved to the supplements allowing you to expand 1B as the points are difficult to see. Lastly, change the colour palette to something that is colour-blind friendly as I couldn’t distinguish the high from low.**

*Response: Moved Figure 1C to supplementary*

**2. Figure 2 – include legend in the figure itself rather than only in the caption. Figure 2B the y-axis title needs to be changed to Proper form.**

*Response: Done* **3. Figure 3 – colour-blind friendly palettes**

*Response: Improved*

**Reviewer #3**

**The manuscript “Plasmodium falciparum parasite prevalence in East Africa: updating data for malaria stratification” is an excellent article presenting methods for utilizing national malaria prevalence survey data for sub-national estimation of parasite prevalence. The spatial and statistical methods are sound and the authors’ integration of data across contiguous countries is fantastic, as malaria transmission is widely known to have great impacts in border regions due to divergence in national control programs. I have few major concerns and other minor critiques to be addressed.**

*Response: We would like to thank reviewer for encouraging comments*

**Major critiques:

The justification for using large parasite prevalence surveys, conducted infrequently, for this work is a bit weak.**

*Response: We disagree that there is a weak justification for this paper in general. The objective was to provide this metric that can be use alongside other input data. Parasite prevalence mapping improve with additional data spatially and temporally and we justify providing this layer give the additional data assembly effort.*

**Most countries now use dhis2 surveillance systems to monitor malaria incidence from all health facilities. Many have also integrated community health worker reporting into this system. This has a much higher spatial resolution and more detailed data. It is not publicly reported, but accessible through NMCPs**

*Response: We agree routine data is available all year round but cover only asymptomatic population accessing health facilities or where community health workers report data covered at the community level. The coverage of community health workers and reporting is still poor at national level. There still issues of pairing this data with parasite prevalence beyond the scope of current study (Page 23)*

**The primary research question is different, as these are clinical cases and do not represent parasite prevalence, but are generally used as a proxy for predicting malaria risk. The authors’ objective was to use the existing prevalence surveys to improve predictive risk maps, while acknowledging the limitations of these surveys.**

**I would like to see a better justification for not using the real time passive surveillance systems to create predictive risk maps, particularly since these surveillance systems typically guide malaria control activities.**

*Response: Agree, the primary research question and endpoint of using clinic data is different.*

*When NMCP use routine passive surveillance data from DHIS2, they estimate malaria incidence (number of confirmed malaria case per population per year) or Test Positivity Rate (TPR) (Kigozi et al., 2019, Alegana et al., 2021) but not parasite prevalence as computed in this manuscript.*

*The objective as correctly pointed out was to use community surveys to generate contemporal risk.*

*The relationship between TPR or incidence with PR is not always linear, can be concave or convex, vary by age and by endemicity. These metrics are also not directly comparable without adjusting for access, immunity or different population structures (Page 23)*

*Real time passive surveillance is more challenging to integrate with PR data and we have outlined these challenges in the discussion (Page 23)*

*Ideal scenario is NMCP to collect these data (conduct survey), have resources for capacity building and lead data analysis in-house, repeatedly. This scenario requires long-term appropriate investment in all areas to generate these tools and use the new maps for decision making. It is not a simple problem to navigate.*

**The authors also fall short on providing justification for the use of these models. It is unclear if this will lead to tools that can be used to inform control programs.**

*Response: The use of Model-Based Geostatistical approaches for mapping malaria risk is not new and has been used in several countries as outlined in the introduction (Page 4 and page 5). The advantage of this approach is the ability to incorporate covariates and measure associated uncertainty related to predictions. There are several reasons for using covariates but mainly for a) prediction in areas with not data due to poor sampling and b) explaining local disease heterogeneity. This are conducted within MBG framework.*

*We have outlined this in the methods and added the element of incorporating uncertainty with predictions to justify use of MBG (page 10 and page 20).*

*Table 1, Figure 4 and Figure 5 are great tools that can be used by national malaria programs*

**This is of particular importance in areas of high prevalence in border regions. Will these methods be shared with NMCPs and how will it be used to guide the national programs? This is mentioned a bit in the discussion, but is not elaborated on and not presented in the introduction as a purpose of the study and potential public health impacts. The findings are great and would really be impactful if integrated into the programs.**

*Response: Both data and products have been shared by the NMCPs and various people from national program are co-authors to this work. Kenya and Tanzania NMCPs will be using these products to conduct mid-term reviews by comparing targets to currents levels of risk. Parasite prevalence will be paired with other intervention data.*

**Minor critiques:

Methods:
With the spatial scope there is incredible heterogeneity in malaria prevalence. The authors age standardized the prevalence measure to PfPR2-10. This is appropriate for high transmission settings where this age groups represents the highest risk group for transmission and identification. In lower transmission settings, this assumption does not hold, and infection reservoirs are primarily older adolescents and young adults. This could skew the results to make areas of low prevalence seem lower than they actually are. Justification for using this measure across varied transmission settings would be helpful.**

*Response: We have added the following justification on page 9 as a justification of mapping PfPR_2-10_*

*“*Parasite prevalence in children aged 2-10 years (*Pf*PR_2-10_) is predictive of other parameters of malaria transmission intensity such as the Entomological Inoculation Rate (EIR) and the Basic Reproduction Rate of Infection (R_0_) (Gething et al., 2014). As such values of *Pf*PR_2-10_ are widely used to model malaria control transmission reduction and the appropriate combinations of available interventions. They have also been used as a variable to predict probability of elimination (Cohen et al., 2010)*.”*

**More information about the RDT to microscopy conversion would be helpful. The use of these measures should also be addressed in the discussion. Both of these tools are diagnostic and not screening tools designed for measuring parasite prevalence. The sensitivity is quite poor in low transmission settings for this purpose and underestimate the prevalence. This could impact the areas that are presented as very low or low risk. Given the findings, there could be substantial misclassification just based on the data collection methods.**

*Response: We have added this limitation in the discussion*

**Results:
It would be helpful to present (in text) the proportion of the total population that is represented in this dataset.**

*Response: included in Table 1*

**In Figure 1 it is clear that most of the data comes from areas of higher population density. Was population density considered as a covariate in the model?**

*Response: Population density was not used as a covariate because that would have introduced endogeneity bias i.e. using covariate in modelling and then using it again to measure population at risk.*

**References**

Alegana, V. A., Atkinson, P. M., Lourenço, C., Ruktanonchai, N. W., Bosco, C., Erbach-Schoenberg, E. Z., Didier, B., Pindolia, D., Le Menach, A., Katokele, S., et al. 2016. Advances in mapping malaria for elimination: fine resolution modelling of Plasmodium falciparum incidence. *Scientific Reports,* 6**,** 29628.

Alegana, V. A., Atkinson, P. M., Wright, J. A., Kamwi, R., Uusiku, P., Katokele, S., Snow, R. W. & Noor, A. M. 2013. Estimation of malaria incidence in northern Namibia in 2009 using Bayesian conditional-autoregressive spatial-temporal models. *Spatial and Spatio-temporal Epidemiology,* 7**,** 25-36.

Alegana, V. A., Okiro, E. A. & Snow, R. W. 2020. Routine data for malaria morbidity estimation in Africa: challenges and prospects. *BMC Med.,* 18**,** 121.

Alegana, V. A., Suiyanka, L., Macharia, P. M., Ikahu-Muchangi, G. & Snow, R. W. 2021. Malaria micro-stratification using routine surveillance data in Western Kenya. *Malaria Journal,* 20**,** 22.

Alegana, V. A., Wright, J., Bosco, C., Okiro, E. A., Atkinson, P. M., Snow, R. W., Tatem, A. J. & Noor, A. M. 2017. Malaria prevalence metrics in low- and middle-income countries: an assessment of precision in nationally-representative surveys. *Malaria Journal,* 16**,** 475.

Banerjee, S., Carling, P. B. & Gelfand, A. E. 2004. *Hierarchical modeling and analysis for spatial data,* London, Chapman & Hall/CRC.

Cohen, J., Moonen, B., Snow, R. & Smith, D. 2010. How absolute is zero? An evaluation of historical and current definitions of malaria elimination. *Malaria Journal,* 9**,** 213.

Gething, P., Battle, K., Bhatt, S., Smith, D., Eisele, T., Cibulskis, R. & Hay, S. 2014. Declining malaria in Africa: improving the measurement of progress. *Malaria Journal,* 13**,** 39.

Giorgi, E., Fronterrè, C., Macharia, P. M., Alegana, V. A., Snow, R. W. & Diggle, P. J. 2021. Model building and assessment of the impact of covariates for disease prevalence mapping in low-resource settings: to explain and to predict. *Journal of The Royal Society Interface,* 18**,** 20210104.

Godwin, J. & Wakefield, J. 2021. Space-time modeling of child mortality at the Admin-2 level in a low and middle income countries context. *Stat Med,* 40**,** 1593-1638.

Kigozi, S. P., Kigozi, R. N., Sserwanga, A., Nankabirwa, J. I., Staedke, S. G., Kamya, M. R. & Pullan, R. L. 2019. Malaria Burden through Routine Reporting: Relationship between Incidence and Test Positivity Rates. *Am J Trop Med Hyg*.

Macharia, P. M., Giorgi, E., Noor, A. M., Waqo, E., Kiptui, R., Okiro, E. A. & Snow, R. W. 2018. Spatio-temporal analysis of Plasmodium falciparum prevalence to understand the past and chart the future of malaria control in Kenya. *Malar. J.,* 17**,** 340.

Noor, A. M., Gething, P. W., Alegana, V. A., Patil, A. P., Hay, S. I., Muchiri, E., Juma, E. & Snow, R. W. 2009. The risks of malaria infection in Kenya in 2009. *BMC Infect Dis,* 9**,** 180.

Noor, A. M., Kinyoki, D. K., Mundia, C. W., Kabaria, C. W., Mutua, J. W., Alegana, V. A., Fall, I. S. & Snow, R. W. 2014. The changing risk of Plasmodium falciparum malaria infection in Africa: 2000-10: a spatial and temporal analysis of transmission intensity. *Lancet,* 383**,** 1739-1747.

Ouma, J., Jeffery, C., Awor, C. A., Muruta, A., Musinguzi, J., Wanyenze, R. K., Biraro, S., Levin, J. & Valadez, J. J. 2021. Model-based small area estimation methods and precise district-level HIV prevalence estimates in Uganda. *PLOS ONE,* 16**,** e0253375.

Plotkin, M., Bishanga, D., Kidanto, H., Jennings, M. C., Ricca, J., Mwanamsangu, A., Tibaijuka, G., Lemwayi, R., Ngereza, B., Drake, M., et al. 2018. Tracking facility-based perinatal deaths in Tanzania: Results from an indicator validation assessment. *PLOS ONE,* 13**,** e0201238.

Snow, R. W., Sartorius, B., Kyalo, D., Maina, J., Amratia, P., Mundia, C. W., Bejon, P. & Noor, A. M. 2017. The prevalence of *Plasmodium falciparum* in sub-Saharan Africa since 1900. *Nature,* 550**,** 515-518.

Tatem, A., Adamo, S., Bharti, N., Burgert, C., Castro, M., Dorelien, A., Fink, G., Linard, C., Mendelsohn, J., Montana, L., et al. 2012. Mapping populations at risk: improving spatial demographic data for infectious disease modeling and metric derivation. *Population Health Metrics,* 10**,** 8.

Tatem, A., Campiz, N., Gething, P., Snow, R. & Linard, C. 2011. The effects of spatial population dataset choice on estimates of population at risk of disease. *Population Health Metrics,* 9**,** 4.
