# Supplementary material for: Plasmodium falciparum parasite prevalence in East Africa: Updating data for malaria stratification
Source: PLOS Glob Public Health. Author manuscript; Available in PMC 2022 Feb 23. (PMC7612417; doi:10.1371/journal.pgph.0000014)
Supplement: Supplementary file 1 [file EMS143660-supplement-Supporting_Information.pdf]

threshold. A NEP close to 100% indicates that PA*Pf*PR_{2-10} is highly likely to be below the threshold *l*; if close to 0%, PA*Pf*PR_{2-10}, is highly likely to be above the threshold *l*; if close to 50%, PAfPR$_{2-10}$, is equally likely to be above or below the threshold *l*, hence corresponding to a high level of uncertainty.

https://doi.org/10.1371/journal.pgph.0000014.g005

Supporting information

S1 Text. Data acknowledgements.
(DOCX)

S2 Text. Covariate selection.
(DOCX)

S3 Text. Spatiotemporal modelling of malaria risk.
(DOCX)

S1 Table. PA*Pf*PR$_{2\text{-}10}$ estimates.
(DOCX)

S1 Fig. Additional maps.
(DOCX)
